# Competition in Early Childhood: A Systematic Review of Developmental Evidence and a Preliminary Ecological Framework

**DOI:** 10.3390/bs16101746

**Published:** 2026-09-24

**Authors:** Sadiye Keleş

**Affiliations:** Early Childhood Education Department, Faculty of Education, Zonguldak Bülent Ecevit University, Zonguldak 67300, Türkiye; skeles@beun.edu.tr

**Keywords:** competition, early childhood education, systematic review, social development, ecological framework, competitive play

## Abstract

Competition remains common but under-theorized in early childhood education (ECE), and its developmental implications are fragmented across distinct research traditions. This systematic review synthesized 56 peer-reviewed empirical studies published between 2000 and 2025 and identified through PsycINFO, Scopus, Web of Science, ERIC, PubMed, and citation searching. Eligible studies examined competitive experiences in children from birth through age 8 across socio-emotional, socio-cognitive, and socio-moral domains. Methodological quality was appraised using the Mixed Methods Appraisal Tool (MMAT), and findings were integrated through a typological narrative synthesis followed by an ecological interpretation. Across heterogeneous experimental, correlational, longitudinal, observational, and qualitative evidence, competition was neither uniformly beneficial nor harmful. Reported developmental findings varied with children’s capacities and relational positioning, the demands and social architecture of competitive activities, and the sociocultural meanings through which competition was interpreted and socialized. Methodological concerns included limited representativeness, variable control of confounding, and sparse direct examination of teacher mediation and cross-cultural mechanisms. Drawing on this synthesis, the review proposes the Safe Zone Framework—Assess, Adapt, and Support—as a preliminary evidence-informed conceptual framework for considering how competitive experiences may be understood, structured, and supported in ECE. The framework re-quires empirical validation before it can be considered a classroom protocol.

## 1. Introduction

Competition is a recurring yet contested feature of early childhood education (ECE), occurring through games, classroom activities, peer comparisons, and reward structures (Sheridan & Williams, 2006; M. Yıldız & Çiftçi, 2025). Within Deutsch’s Cooperation–Competition Theory, cooperation and competition are distinguished by the structure of interdependence: positive interdependence occurs when individuals’ goals are mutually facilitated, whereas negative interdependence occurs when one individual’s goal attainment reduces another’s likelihood of success (Deutsch, 2012). From this perspective, competition does not constitute a single pedagogical condition; its developmental implications may vary according to how goals, participation, rewards, and peer comparison are structured. This distinction is particularly important in ECE, where classroom practices organize not only achievement opportunities but also children’s access to participation, peer interaction, and socio-emotional, socio-cognitive, and socio-moral learning experiences. Elimination-based activities such as musical chairs illustrate one specific competitive structure: as children are progressively removed from the activity, continued participation becomes contingent on outperforming peers, potentially restricting some children’s access to subsequent interaction and practice. Other competitive formats, however, may organize participation and interdependence differently and therefore need not produce the same developmental consequences. The central pedagogical question is thus not whether competition is inherently beneficial or harmful, but how its developmental implications vary with children’s capacities and relationships, the demands and social organization of competitive activities, and the broader meanings attached to competition. Addressing this question requires an approach that neither uncritically promotes nor categorically dismisses competition in ECE.

Competition in developmental and educational research is not a unitary construct but encompasses several related yet conceptually distinct phenomena. At least five levels can be distinguished. First, dispositional or trait competitiveness refers to a relatively stable tendency to seek comparative advantage or to outperform others (Helmreich & Spence, 1978). Second, perceived competitive climate concerns the extent to which an environment is experienced as emphasizing comparison, ranking, or outperforming peers (Murayama & Elliot, 2012). Third, experimentally or pedagogically structured competition refers to situations in which goals, rewards, or resources are organized through negative interdependence, such that one participant’s success reduces another’s likelihood of attaining the same outcome (Johnson & Johnson, 2009). Fourth, competition orientation refers to relatively enduring ways of approaching, interpreting, or disengaging from competitive situations. Rather than reflecting a single continuum, it is multidimensional and may include self-developmental, other-referenced or hypercompetitive, competition-avoidant, and competition-uninterested orientations (Paquette et al., 2013; Orosz et al., 2018). Finally, competitive outcomes refer to the consequences or endpoints generated within competitive situations, such as winning, losing, exclusion, blocked goal attainment, or resource allocation. These constructs are related but not interchangeable: they locate competition respectively within the child, the perceived environment, the structure of an activity, the orientation of striving, or the resulting experience. In this review, competition is therefore used as an umbrella concept for empirical studies in early childhood that explicitly examine one or more of these dimensions, while preserving the distinction between them during synthesis. Evidence from older children, adolescents, or adults is used only to clarify the broader conceptual landscape and is not treated as direct evidence of competition in ECE (Rudolf & Lee, 2023; Fülöp et al., 2025; Yang et al., 2025).

Empirical findings further underscore the heterogeneity of competition in early childhood. Competitive conditions do not produce uniform effects across developmental domains or activity structures. For example, brief competitive interaction did not enhance 4-year-old children’s reasoning about others’ differing desires, whereas cooperative interaction did (Jin et al., 2017). In contrast, intergroup competition has been associated with increased prosocial behaviour toward in-group members under some conditions, with effects differing by age (Zhu et al., 2015). Similarly, children’s resource-allocation decisions following competition vary according to whether competition occurs between individuals or groups, whether children win or lose, and their developmental level (Xiao et al., 2024b). Together, these findings suggest that competition cannot be assigned a single developmental valence: its implications depend on the form of interdependence, the social target of competition, the outcome experienced, and the developmental process under consideration.

Evidence from broader educational and adult studies offers additional conceptual support for this context-sensitive interpretation, although it should not be treated as direct evidence about ECE. Reward-intensive competitive environments, for example, have been associated with lower intrinsic motivation and satisfaction among university students (Hanus & Fox, 2015/2018), while individual differences such as guilt proneness have been linked to preferences for competitive versus non-competitive pathways to achievement in adult samples (Haran et al., 2024). Such adjacent evidence reinforces the importance of distinguishing competitive structures from individual orientations, while also highlighting the need to establish these relationships specifically within early childhood populations.

Previous syntheses have addressed important but relatively discrete dimensions of competition in childhood and ECE. Green and Rechis (2006) reviewed children’s cooperative and competitive interactions in limited-resource situations, emphasizing negotiation, conflict, and the development of social competence. More recently, Tsiakara and Digelidis (2021) synthesized evidence on competitive behaviour in the preschool years, highlighting variation associated with age, gender, and group composition. Related work has also conceptualized competition and cooperation as potentially coexisting dimensions of preschool experience (Sheridan & Williams, 2006). These contributions establish that competitive behaviour emerges early and is shaped by both child and contextual characteristics; however, they do not provide an integrated account of how different competitive structures intersect with developmental capacities, peer relations, activity organization, and sociocultural context across socio-emotional, socio-cognitive, and socio-moral domains. The present review addresses this gap by moving beyond the classification of competitive behaviours or correlates to examine the configurations of conditions under which competition is associated with different developmental experiences and outcomes in ECE.

### 1.1. Ethical and Pedagogical Decision-Making Gap

In ECE, competition may emerge around access to adult attention, limited resources, recognition, or relative advantage among peers (Weinberger & Stein, 2008; Tsiakara & Digelidis, 2015). Within such situations, children may use verbal and nonverbal strategies to negotiate advantage, while competitive interactions may influence peer positioning and status relations. Competitive situations may also recruit socio-cognitive strategies as children anticipate opponents’ actions and use mental-state understanding in the pursuit of incompatible goals (Priewasser et al., 2013). Competition therefore cannot be evaluated solely in terms of winning and losing: depending on how it is structured and mediated, the same competitive situation may involve opportunities for motivation, strategic thinking, peer interaction, participation, or exclusion.

This complexity creates an ethical and pedagogical decision-making problem. Although existing studies document particular developmental correlates of competition, they offer limited integrated guidance concerning when, how, and under what conditions competitive experiences may support or constrain children’s participation and development. This concern is consistent with a rights-based understanding of participation, in which children’s involvement entails not only opportunities to express their perspectives but also having those perspectives given meaningful consideration in matters affecting their experiences (Lundy, 2007). In ECE specifically, research on children’s participation has also shown that children are comparatively underrepresented as direct informants, reinforcing the need to attend more explicitly to their perspectives in pedagogical decision-making (Correia et al., 2019). The central ethical issue is therefore not competition per se, but how competitive structures distribute opportunities to participate, persist, interact, and succeed among children with different developmental and social resources. An integrative synthesis connecting developmental evidence with pedagogical and contextual conditions is needed to move beyond simple pro- versus anti-competition positions and toward more differentiated decisions about competition in early childhood settings.

### 1.2. Current Study

The present systematic review synthesizes empirical research published between 2000 and 2025 to examine how competition has been conceptualized, structured, and examined in relation to socio-emotional, socio-cognitive, and socio-moral development in early childhood. Specifically, the review addresses four questions: (1) What forms and structures of competition have been examined in early childhood research? (2) What socio-emotional, socio-cognitive, and socio-moral findings are reported in relation to these forms of competition? (3) How do methodological and contextual characteristics shape the interpretation of these associations? (4) What patterns emerge when the evidence is considered across child/interactional, pedagogical/activity, and broader sociocultural contexts?

Beyond summarizing domain-specific findings, the review examines whether apparently divergent findings can be understood in relation to the conditions under which competition occurs. Bronfenbrenner’s ecological perspective conceptualizes development as emerging through reciprocal relations between the developing person and interconnected environmental contexts (Bronfenbrenner, 1979), while later bioecological formulations emphasize the dynamic interplay among process, person, context, and time (Bronfenbrenner & Morris, 2006). Informed by this perspective, the present review uses child/interactional, pedagogical/activity, and sociocultural contexts as an adapted interpretive heuristic rather than as direct equivalents of Bronfenbrenner’s nested systems. The resulting synthesis provides the basis for a preliminary evidence-informed framework for the pedagogical structuring of competition in ECE, which is proposed as a conceptual contribution requiring empirical validation.

## 2. Methods

### 2.1. Search Strategy and Eligibility Criteria

A systematic review of the literature was conducted using Web of Science Core Collection, Scopus, ERIC, PubMed, and PsycINFO. During revision, the database searches were rerun in full rather than incrementally supplemented. The revised search broadened the terminology used to identify competition-related research, added PsycINFO, and applied database-specific search fields and filters. All searches were last run on 16 September 2026 and were restricted to publications from 2000 to 2025.

Three conceptual search blocks were combined using Boolean operators. The competition block included competition, competitive, rivalry, rival*, competitive play, competitive game*, social comparison, rank*, zero-sum, winning, losing, win, peer status, and resource competition. The developmental and social-outcome block included social, emotion*, aggression, motivation, self-regulation, prosocial*, cooperation, fairness, resource allocation, moral reasoning, theory of mind, ToM, deception, sharing, and peer*. The age/population block included early childhood, child*, preschool*, kindergarten*, toddler*, young child* and primary school. Search syntax was adapted to the indexing structure and search fields of each database.

Database-specific field selections and filters were applied as follows: Web of Science was searched using the Topic field (TS=), with records restricted to articles published in English and subject categories outside the scope of the review excluded; Scopus was searched using TITLE-ABS-KEY, with document type restricted to Article, source type to Journal, and language to English, and unrelated subject areas excluded; ERIC was restricted to records published between 2000 and 2025 and to peer-reviewed sources; PubMed search terms were restricted to Title/Abstract and age filters were used to capture child populations potentially overlapping the review age range; and PsycINFO was searched in Title/Abstract (XB) with source type restricted to Article. The final rerun yielded 9127 records from Web of Science, 8171 from Scopus, 800 from ERIC, 1860 from PubMed, and 2192 from PsycINFO, for a total of 22,150 database records before deduplication. Full database-specific search strings, field codes, limits, subject exclusions, search dates, and retrieval counts are reported in Supplementary Table S1.

Studies were eligible for inclusion if they (a) were published between 2000 and 2025; (b) reported original empirical research involving children from birth through 8 years of age; (c) examined competition, competitive situations, competitive play, competitive games, or related competitive structures as a focal variable or condition and allowed the role of competition to be distinguished from co-occurring intervention or activity features; (d) reported findings relevant to at least one of the three domains examined in the review: socio-emotional, socio-cognitive, or socio-moral development; (e) were published as peer-reviewed journal articles; and (f) had a full text available in English. Citation searching, including examination of the reference lists of relevant and eligible studies, was additionally conducted to identify potentially eligible records not retrieved through the database searches. Eligibility was defined developmentally rather than by educational placement; therefore, studies involving children through age 8 were eligible even when participants were enrolled in the early primary grades, provided that the substantive developmental criteria were met. Studies were eligible when all child participants were younger than 9 years (i.e., within the birth-through-8-years developmental range). Studies including participants aged 9 years or older were excluded, irrespective of whether age-group-specific findings were reported separately.

Studies centered on children with diagnosed neurodevelopmental or clinical conditions, including studies comparing a clinical or neurodevelopmental group with typically developing peers, were excluded because the review aimed to synthesize competition within general developmental and educational contexts. Sources cited elsewhere in the manuscript for theoretical, methodological, or contextual framing were not considered part of the evidentiary corpus unless they independently met the eligibility criteria. Reviews, meta-analyses, books, book chapters, conference proceedings or abstracts, dissertations, theses, repository-only manuscripts, and other forms of grey literature were excluded. Studies focused primarily on organized sport or athletic competition were also excluded because the review concerned competition within developmental and educational contexts rather than sport-specific performance environments. No restrictions were placed on methodological design; experimental, observational, correlational, longitudinal, and qualitative studies were eligible when they met the substantive criteria.

### 2.2. Study Selection and Reviewer Procedures

Records retrieved from the databases were combined in Microsoft Excel. Duplicate records were identified using title, author, publication year, and DOI information and were manually verified before removal. Titles and abstracts were screened against the predefined eligibility criteria, after which potentially eligible reports underwent full-text assessment. Reasons for exclusion at the full-text stage were recorded and are summarized in the PRISMA flow diagram (Page et al., 2021). As this was a single-author systematic review, title and abstract screening, full-text eligibility assessment, data extraction, methodological appraisal, and synthesis were conducted by the author. Independent duplicate screening and duplicate data extraction were not undertaken. To provide an independent check of eligibility decisions, 20 of the 80 reports assessed at full text were randomly selected for duplicate review. The author and an independent researcher separately evaluated each selected report against two central eligibility criteria: (a) whether all child participants were younger than 9 years and (b) whether competition constituted a focal construct, condition, or experience rather than an incidental contextual feature. Agreement was complete for the age criterion (20/20, 100%) and was reached for 17 of 20 reports (85%) for the competition-focus criterion. Three criterion-level disagreements were identified. Two did not affect the overall eligibility decision because both reviewers independently classified the reports as age-ineligible. The remaining disagreement concerned Herrmann et al. (2019); following re-examination of the full report against the prespecified eligibility criteria, the study was retained because competitive partner choice constituted a focal experimental construct. Overall eligibility agreement prior to resolution was 19/20 (95%).

### 2.3. Methodological Quality Appraisal

Methodological quality was appraised using the Mixed Methods Appraisal Tool (MMAT; Hong et al., 2018). Each study was first classified according to its methodological design and then assessed against the five design-specific MMAT criteria, following the two initial screening questions. Judgments were recorded as Yes, No, or Can’t tell. For quantitative studies, an outcome-completeness threshold of at least 80% was applied. No overall numerical quality scores were calculated; instead, criterion-level judgments were retained to identify specific sources of methodological uncertainty. The appraisal informed the synthesis qualitatively. Findings from studies with substantial concerns regarding measurement, attrition, confounding, or experimental control were interpreted more cautiously and were not treated as equivalent to findings supported by methodologically stronger designs. Study-level judgments are reported in Table A1.

### 2.4. Data Analysis and Synthesis

Given substantial heterogeneity in study designs, operationalizations of competition, outcome constructs, and measures, a meta-analysis was not undertaken. Instead, a typological narrative synthesis was used, drawing on typological analysis (Hatch, 2023) and established guidance for narrative synthesis in systematic reviews (Popay et al., 2006). Data were extracted using a structured form covering publication year, country/context, participant characteristics, study design, competition measure or task, developmental domain(s), and principal competition-related findings. When a study reported findings relevant to more than one developmental domain, all eligible domain-specific findings were retained for synthesis.

The synthesis proceeded in two stages. First, findings were organized into socio-emotional, socio-cognitive, and socio-moral domains; studies could contribute to more than one domain. Within each developmental domain, studies were additionally differentiated according to the primary form in which competition was conceptualized or operationalized: dispositional competitiveness, perceived competitive climate, competitive situation/structure, competition orientation, or competitive outcomes/experiences. Where studies involved experimentally or naturally occurring competitive situations, the social structure of competition (e.g., interpersonal, intergroup, resource-based, or peer-status competition) was also retained during synthesis. Research contexts were also differentiated to distinguish structured experimental or task-based evidence from naturalistic educational or peer contexts, survey/questionnaire-based evidence, structured behavioral-assessment contexts, and qualitative/multimethod naturalistic evidence. Experimental, correlational, longitudinal, observational, and qualitative evidence were distinguished rather than treated as inferentially equivalent, and null or contradictory findings were retained. Second, recurring patterns were interpreted through an ecological heuristic comprising child/interactional, pedagogical/activity, and sociocultural contexts. These contexts functioned as an interpretive structure rather than as predetermined coding categories. MMAT judgments informed the interpretation of recurring patterns, with findings affected by important methodological concerns treated more cautiously. The Safe Zone Framework was not used as an a priori coding scheme. Following the developmental and ecological synthesis, recurring patterns concerning individual variability, competitive activity structure, and emotional, relational, and socio-moral demands were examined iteratively and conceptualized as Assess, Adapt, and Support. Thus, the framework represents a subsequent interpretive product of the synthesis rather than a classification imposed on the included studies.

Study characteristics and principal findings were tabulated to facilitate structured cross-study comparison. No statistical data transformations, imputations, or conversions were performed for the narrative synthesis. No formal assessments of reporting bias or certainty of the body of evidence were undertaken. Because the review employed a narrative synthesis across heterogeneous study designs and outcome constructs, no common effect measure was specified or calculated across studies. Where available, study-specific statistical results were interpreted in the form reported by the original authors and are summarized in Supplementary Table S3.

## 3. Results

Table 1 summarizes the 56 studies included in the review. The included studies represented considerable methodological and contextual diversity, encompassing experimental, correlational, longitudinal, observational, and qualitative designs. Competition was operationalized through dyadic and intergroup games, resource-allocation tasks, cognitively mediated strategic paradigms, and questionnaire-based measures, underscoring the heterogeneity of the evidence base.

### 3.1. Study Selection

The database searches identified 22,150 records, and an additional six records were identified through citation searching. After removal of 7398 duplicate records, 14,752 database-derived records were screened by title and abstract, of which 14,676 were excluded. Seventy-six reports were sought for retrieval, and two could not be retrieved, leaving 74 database-derived reports for full-text eligibility assessment. All six reports identified through citation searching were retrieved and assessed for eligibility. Across both identification pathways, 80 reports were assessed at full text. Twenty-four reports were excluded according to the predefined eligibility criteria, including 21 from the database-search pathway and three from citation searching. Consequently, 56 studies met the inclusion criteria and were included in the review (Figure 1).

### 3.2. Methodological Quality of the Evidence Base

Criterion-level appraisal indicated substantial methodological variation across the 56 included studies. The qualitative studies generally showed good coherence between their research aims, data sources, analytic procedures, and interpretations. Several incorporated multiple data sources or triangulation, although their small and context-specific samples constrained transferability. For example, Sheridan and Williams (2006) combined interviews, drawings, and observations and explicitly used triangulation, whereas Kalkman et al.’s (2017) ethnographic analysis centred primarily on a single focal video episode. Among randomized quantitative studies, baseline comparability and outcome completeness were generally adequate, whereas randomization procedures and outcome-assessor blinding were often insufficiently reported. Intervention exposure and measurement reliability were also not uniformly strong. Eriksson et al. (2021), for example, reported substantial variation in participation across gaming sessions and behavioral tasks, together with poor reliability for competitive-behavior coding. 

Non-randomized quantitative studies generally employed appropriate measures and retained adequate outcome data, but representativeness was frequently limited and control of confounding varied considerably. Particularly consequential concerns included fixed-order comparisons, performance-determined win–loss outcomes, confounding between competitive context and interaction structure, common-informant measurement, and selective attrition. Accordingly, findings affected by these limitations were interpreted cautiously and treated as supportive rather than stand-alone evidence when identifying recurring cross-study patterns.

### 3.3. Forms and Operationalizations of Competition Across Studies

The included studies did not examine a single, uniform construct of competition. Consistent with the conceptual distinctions outlined in the Introduction, the evidence base encompassed dispositional competitiveness, perceived competitive climate, competitive situations/structures, competition orientations, and competitive outcomes or experiences. These categories were analytically distinguished because they locate competition at different levels: as a relatively enduring individual tendency, a perceived environmental characteristic, a socially structured activity condition, an orientation toward competitive engagement, or an outcome generated within a competitive episode. Studies addressing more than one dimension were classified according to their primary operationalization while secondary competitive features were retained during synthesis.

The largest portion of the evidence concerned competitive situations or structures, including experimentally structured interpersonal and intergroup competition, resource-based competition, strategic competitive tasks, and naturally occurring peer competition. A smaller set of studies examined *competition orientations*, including task-oriented and other-referenced competition or competitive social-value choices. Other studies focused primarily on *competitive outcomes and experiences*, such as winning, losing, relative status, exclusion, or post-competition allocation. Evidence concerning stable dispositional competitiveness or sustained perceived competitive climates was comparatively limited.

### 3.4. Socio-Emotional Finding

Socio-emotional findings were heterogeneous, indicating that competition was neither uniformly harmful nor beneficial. Tsiakara and Digelidis (2015) found similar perceived performance and satisfaction across competitive and noncompetitive conditions, whereas Eriksson et al. (2021) reported greater enjoyment of cooperative games and lower enjoyment of competition following loss. The latter evidence warrants caution because participation varied across sessions and competitive-behavior coding showed poor reliability. Responses also varied between children. Hughes et al. (2001) found more negative reactions to competitive threat among children identified as hard-to-manage, with earlier violent pretend play predicting later negative behavior.

Qualitative evidence similarly showed that losing could elicit aggression, particularly among children with lower peer acceptance, while also prompting coping and re-engagement strategies (Emen Parlatan & Sığırtmaç, 2022). Competition orientation was also associated with adjustment. Self-regulation was positively associated with task-oriented competition (Bayındır et al., 2022), whereas task-oriented competition showed weak negative associations with several anxiety dimensions (S. Yıldız & Sak, 2024). These cross-sectional associations do not establish causal effects.

### 3.5. Socio-Cognitive Findings

Socio-cognitive findings indicate that competitive context can shape the deployment of emerging social-cognitive capacities rather than uniformly enhance them. Cooperation produced stronger performance than competition in reasoning about diverse desires and visual perspectives (Jin et al., 2017; P. Li et al., 2019).

Younger 4-year-olds also deployed ToM more successfully for cooperative than competitive goals (Tsoi et al., 2021), while Domberg et al. (2018) found fewer and more self-serving arguments under competition, particularly among older children. Within competitive contexts, however, more developed social cognition was associated with greater sensitivity to opponents’ goals and more strategic behavior. False-belief and related mental-state understanding were associated with strategic poaching, competitive lying, and understanding competitive sabotage (Priewasser et al., 2013; Sai et al., 2020; Priewasser et al., 2025). Prodan et al. (2023) similarly found that ToM and executive functions were associated with strategic deception toward peer opponents, with children deceiving familiar peers less than unfamiliar peers. Yet competence did not necessarily translate into action: Proft et al. (2021) found that 3-year-olds could represent competing desires but did not consistently use this understanding in their competitive choices. Smith and LaFreniere (2013) further demonstrated a clear developmental progression in strategic behavior within interpersonal competition. In a competitive card-guessing task, 4-year-olds were largely non-strategic and rarely successful, whereas 6-year-olds showed increasing use of deceptive strategies and 8-year-olds were substantially more successful and more likely to demonstrate an explicit understanding of their opponent’s expectations. Success rates increased from 11% among 4-year-olds to 49% among 6-year-olds and 76% among 8-year-olds, indicating a marked age-related increase in children’s ability to manage information strategically during competition. Developmental differences were also evident in context-sensitive social decision-making. From around age five, children selected task-relevant capable partners for cooperation while avoiding them as competitors (Grueneisen et al., 2024), while older children showed greater sensitivity to informants’ competing interests in selective-trust judgments (M. Zhang & Sylva, 2021). Together, these findings indicate developmental variation in how children apply socio-cognitive capacities to divergent social goals.

### 3.6. Socio-Moral Findings

Socio-moral findings suggest that competition does not simply weaken prosociality, but changes how children balance fairness, self-interest, and relational concerns. Competition reduced generosity in some contexts: Pappert et al. (2017) found fewer prosocial choices under costly disadvantageous inequality, while Toppe et al. (2019) reported less sharing with uninvolved third parties after competitive than cooperative play. However, Toppe et al. (2021) found no effect on prosociality or ingroup bias, indicating that spillover is not uniform. Fairness judgments were shaped by context and outcome. After interpersonal competition, children increasingly favored merit when they won but equality when they lost, whereas intergroup competition elicited more equal and less own-side-biased allocations (Xiao et al., 2024b). Xiao et al. (2024a) further showed that older children balanced merit against ingroup affiliation while expecting teachers to allocate according to merit, revealing a distinction between moral norm knowledge and enacted choice. Zhao et al. (2025) similarly found a developmental shift from self-favoring toward ingroup-favoring allocations in competition. Socio-moral functioning also extended beyond allocation. Children emphasized fair play and equal opportunity (Sheridan & Williams, 2006), while reconciliation after competitive conflict helped repair peer relationships (Roseth et al., 2011). Overall, competition appears to reorganize the relative weight of equality, merit, self-interest, group loyalty, and relational obligations rather than uniformly promoting or undermining morality.

### 3.7. Ecological Synthesis of Findings

Across the reviewed studies, cross-domain patterns were interpreted across three interconnected contexts: child/interactional, pedagogical/activity, and sociocultural (Table 2). These categories are used as an adapted ecological heuristic rather than as direct equivalents of Bronfenbrenner’s micro-, meso-, and macrosystems. At the child/interactional context, competitive responses varied with children’s developmental capacities, relational positioning, and the characteristics of those with whom they competed. Socio-cognitive capacities were particularly relevant to strategic behavior. False-belief understanding and related mental-state capacities were associated with strategic competitive behavior (Priewasser et al., 2013; Sai et al., 2020; Prodan et al., 2023), while Smith and LaFreniere (2013) demonstrated a marked developmental progression in tactical deception: older children were increasingly able to inhibit informative cues, mislead an opponent, and reason explicitly about the opponent’s expectations. Regulatory capacities, however, did not operate uniformly across social contexts; Huyder and Nilsen (2012) found that inhibitory control predicted fewer competitive behaviors in a cooperative context but not behavior within competition itself. Competitive responses also varied with gender and relational position. Benenson et al. (2001) showed that the expression of competitive behavior differed according to both gender and group size, while Ensor et al. (2011) identified gender differences in problem behavior during competitive play with friends. Peer acceptance, dominance, familiarity, reconciliation, and opponents’ behavior further differentiated children’s responses (Roseth et al., 2011; Emen Parlatan & Sığırtmaç, 2022; Prodan et al., 2023). Taken together, these findings indicate that developmental characteristics do not function as stable indicators of competitive “readiness”; their relevance emerges in relation to particular opponents, relationships, and competitive demands.

At the pedagogical/activity context, competition varied substantially in both the demands imposed by the activity and the social architecture through which competitive relations were organized. Studies included performance- or action-based activities requiring speed, dexterity, coordination, or task execution; cognitively mediated activities requiring deception, perspective taking, reasoning, or strategic anticipation; and distributive activities involving sharing, merit, equality, or group interests. These demands were further differentiated by interpersonal versus intergroup organization, group size and composition, goal and reward interdependence, public or private win–loss outcomes, future partner choice, and adult intervention. Benenson et al. (2001), for example, showed that the same competitive game elicited different behavioral and emotional patterns in dyads and tetrads, illustrating that group size itself can alter the social experience of competition. Herrmann et al. (2019) demonstrated another form of social architecture: by age 8, children increased generosity when their behavior could affect subsequent partner selection, indicating that competition may operate through reputational and partner-choice processes rather than through direct win–loss opposition alone. Intergroup competition introduced still different demands. Xiao and Li (2025) showed that competitive outcome and group status altered subsequent allocation priorities, with age-related shifts toward equality after winning and generosity toward in-group collaborators after losing. Teacher involvement also formed part of the activity ecology. In naturally occurring aggressive competitive conflicts, Roseth et al. (2008) found that teacher intervention altered the conflict–peacemaking sequence rather than producing uniformly beneficial or detrimental outcomes: intervention increased separation but was also associated with subsequent reconciliation among separated children. Thus, the developmental significance of competition appears to depend on configurations of task demands, goal relations, group structure, outcome status, reputational consequences, and adult mediation rather than on competition as a unitary activity condition.

At the sociocultural context, the evidence remained more limited but became more differentiated when cultural background, culturally situated knowledge, and emotional expression were considered separately. Strand et al. (2015) reported that the association between competitive social values and behavioral adjustment differed across cultural-background groups. Savage et al. (2023), however, provided an important boundary finding: although competitive social-value choices varied across family cultural-background groups, neither social-value orientation nor its interaction with cultural background was associated with teacher-rated prosocial or problem behavior. Direct cross-cultural evidence further suggested that culture may shape the expression of competitive experience. Mui et al. (2017) found that children’s smiling following winning and losing varied as a function of both social and cultural context, with the effect of playing socially on smile intensity differing between Chinese and Dutch children. Complementing these findings, Goldvicht-Bacon and Diesendruck (2016) showed that 5-year-old Israeli children adjusted strategic choices in a competitive coordination task according to whether an opponent was presented as belonging to the same or a different cultural group, suggesting that assumptions about culturally shared knowledge can enter into competitive reasoning. Qualitative and family-level evidence further suggested possible socialization pathways through parenting practices, sibling comparison, and competition-related narratives (Guimond et al., 2012; S. Yıldız & Sak, 2024; Liu et al., 2025). These findings do not establish that culture consistently moderates the developmental consequences of competition. Rather, they indicate that culturally situated meanings, expectations, and expressive norms may shape how competitive situations are interpreted and enacted.

Taken together, the ecological synthesis indicates that competition is best understood as a configurational rather than an isolated developmental condition. Competitive responses emerge from the interaction between *children’s developing capacities* and *relational positioning*, the *social architecture* and *outcome structure of competitive activities*, and the *sociocultural meanings* and *expectations through which those activities are interpreted*. Across these contexts, the same nominally competitive situation may therefore carry different developmental demands and meanings depending on who is competing, with whom, under what task and group conditions, and within which social and cultural frame.

Qualitative evidence from China further identified possible socialization pathways. Children simultaneously valued winning, friendship, and enjoyment, while sibling comparison and competition-themed picture books were described as influences on their understandings and enactments of competition (Liu et al., 2025). Family-level evidence also linked parenting practices with children’s behavior in competitive peer situations and with particular competitive orientations, although these findings provide indirect rather than direct evidence of sociocultural influence (Guimond et al., 2012; S. Yıldız & Sak, 2024). Overall, the sociocultural evidence suggests that competitive meanings and responses may be shaped by culturally situated expectations and socialization processes, but the available studies do not establish that culture consistently moderates developmental outcomes. Direct cross-cultural studies using comparable competitive structures and measures are therefore still needed. Taken together, the evidence suggests that competition is best understood as an ecologically contingent experience: developmental patterns vary with children’s capacities and relational positioning, the demands and social architecture of competitive activities, and the broader meanings through which competition is socialized and interpreted.

Across the domain-specific and ecological synthesis, three recurring evidence patterns were identified concerning child-level variability and relational positioning, the structure of competitive activities, and the emotional, relational, and socio-moral demands of competition. These patterns were subsequently conceptualized as Assess, Adapt, and Support. Table 3 summarizes the study-level evidentiary basis, boundary findings, and MMAT-informed interpretation underlying each component of the preliminary Safe Zone Framework.

## 4. Discussion

### 4.1. Reframing Competition as a Context-Dependent Developmental Process

The principal contribution of this review is a shift in the unit of explanation. The accumulated evidence does not support treating competition itself as a sufficiently informative developmental condition. Rather, the same nominally competitive situation may acquire different developmental meanings depending on the child, the relationship in which competition occurs, the demands of the activity, and the wider social meanings attached to competitive success and failure (Fülöp, 2009).

Accordingly, the relevant analytical question is not whether competition produces positive or negative outcomes, but under what configurations of conditions particular developmental patterns become more or less likely (Gevaux & Nilsen, 2026).

This distinction is consequential for early childhood research because much of the literature has approached competition through categorical contrasts: competition versus cooperation, task-oriented versus other-referenced competition, or interpersonal versus intergroup competition. These distinctions remain theoretically useful, but the present synthesis indicates that they capture only part of the developmental process. No single competitive form consistently mapped onto a single class of outcomes (see also Fülöp et al., 2025). Instead, its meaning changed as goal structure intersected with developmental capacities, peer relations, task demands, grouping arrangements, and outcome structures. The implication is not that existing classifications are incorrect, but that they are relationally and developmentally incomplete when considered in isolation.

This interpretation both converges with and extends previous syntheses. Green and Rechis (2006) emphasized negotiation, conflict, and social competence in children’s cooperative and competitive interactions, while Tsiakara and Digelidis (2021) highlighted variation associated with age, gender, and group composition in preschool competition. The present review supports their broader conclusion that competitive behavior is context-sensitive, but extends it by showing that no single competitive form maps consistently onto socio-emotional, socio-cognitive, or socio-moral outcomes. Instead, developmental patterns appear to emerge from configurations involving children’s capacities and relationships, activity structure, and sociocultural meaning.

This perspective also offers a more developmentally specific extension of Social Interdependence Theory. Positive and negative interdependence identify how participants’ goals are structured (Deutsch, 2012; Johnson & Johnson, 2009), but young children must still perceive, interpret, and regulate those goal relations through developing socio-cognitive and regulatory capacities. The significance of negative interdependence therefore cannot be inferred from goal opposition alone. For example, the relevance of inhibitory control differed across cooperative and competitive contexts, while mental-state understanding could support strategically competitive behavior when goals were incompatible. These contrasting patterns suggest that developmental capacities do not operate as uniformly protective resources; their function depends on the social goals within which they are mobilized (Huyder & Nilsen, 2012; Priewasser et al., 2013; Sai et al., 2020; Prodan et al., 2023). The contribution of the present synthesis is therefore to place goal interdependence within a broader developmental ecology rather than to treat it as a sufficient explanation of competitive experience.

### 4.2. From Competitive Forms to Competitive Configurations

The ecological interpretation developed in this review differs from a conventional catalogue of child, task, and cultural variables. Its explanatory value lies in treating these dimensions as configurational rather than additive. A child does not encounter “competition” independently of an opponent, task, reward structure, peer group, or history of relationships. Similarly, an activity does not impose a fixed competitive meaning independent of children’s capacities or the norms through which winning and losing (e.g., perceptions of success and failure) are interpreted.

This becomes particularly important at the child/interactional level. Concepts such as self-regulation, ToM, or peer acceptance are often treated as individual predictors, yet the reviewed evidence suggests that their relevance emerges in interaction. A child’s regulatory capacity may matter differently depending on whether goals are shared or opposed; strategic social cognition may facilitate either coordination or interference depending on the social objective; and responses to losing may be shaped by relational standing within the peer group (Roseth et al., 2011; Emen Parlatan & Sığırtmaç, 2022). Developmental characteristics should therefore not be translated into a generalized notion of “competition readiness.” A more defensible interpretation is that competition recruits developmental capacities differently under different relational conditions.

The same argument applies to activity structure. What is often labelled a competitive task may simultaneously vary in performance demands, public comparison, reward contingencies, elimination, group membership, opponent characteristics, and opportunities for continued participation. These features create different social architectures even when the overarching goal remains to outperform another person or group. The pedagogical relevance of this distinction is substantial: competition is not a single instructional technique that can be judged independently of its design. Rather, it is a modifiable arrangement of goals, relations, tasks, and outcomes. This interpretation is consistent with broader ECE research showing that classroom experiences emerge from the interaction between pedagogical practices and structural conditions rather than from isolated instructional features. In multicultural early childhood settings, for example, supportive teacher–child and peer interactions, child engagement, grouping arrangements, and teacher–family relationships have been identified as interconnected contributors to classroom climate (Khalfaoui et al., 2021).

The limited sociocultural evidence further suggests that the meaning of competitive orientation cannot be separated entirely from socialization (Fülöp, 2004). Broader school-based evidence similarly suggests that competitive emphasis may extend beyond the immediate classroom, with teachers perceiving a focus on performance and social comparison across multiple levels of the educational ecology, including parents, school leaders, and governing bodies (Goegan & Daniels, 2022). Particularly important is the indication that similar levels of competitive values may be associated with different forms of adjustment across cultural-background groups. This shifts attention away from asking which cultures are “more competitive” toward the more defensible question of what competition signifies and how it functions within different social environments. At the same time, the evidence base is not sufficient to attribute between-country variation to culture itself. Sociocultural context therefore represents an important explanatory dimension of the framework, but currently the least empirically developed one. Taken together, the ecological contribution of this review lies in a single proposition: the developmental meaning of competition emerges from configurations of conditions rather than from competition as an isolated exposure. This is what becomes visible through the ecological lens that is less apparent when competition is classified solely by goal orientation, interpersonal structure, or task form.

### 4.3. From Ecological Synthesis to the Preliminary Safe Zone Framework

The Safe Zone Framework is proposed as a preliminary evidence-informed framework for the pedagogical consideration of competition in early childhood settings. It is intended for situations in which educators deliberately incorporate competitive activities or in which competition emerges spontaneously within children’s classroom and play experiences; it does not imply that competition should be introduced where it is otherwise absent. Its purpose is not to determine whether competition should be used, but to organize pedagogical attention when competition is already present. The framework translates the ecological synthesis into three interrelated principles—Assess, Adapt, and Support—that address how competitive experiences may be understood, structured, and responded to. These principles have not been evaluated as an integrated classroom intervention and should therefore be regarded as provisional rather than as a validated protocol.

Assess concerns understanding how children experience a competitive situation rather than classifying them according to presumed vulnerability or “readiness.” The review indicates that responses vary with developmental capacities, competition orientation, relational positioning, goal structure, opponent behavior, and child characteristics such as gender (e.g., Q. Zhang et al., 2025). Accordingly, Assess emphasizes responsive observation of children’s emotional, cognitive, behavioral, and relational responses rather than diagnostic profiling, fixed developmental thresholds, or standardized judgments of suitability for competition.

Adapt reflects the finding that competition is not a unitary activity condition. Competitive experiences varied with task demands and social architecture, including goal interdependence, interpersonal versus intergroup organization, grouping, peer composition, reward contingencies, and win–loss structures (Weinberger & Stein, 2008; Zhu et al., 2015; Xiao et al., 2024b). Adapt therefore treats competitive activities as modifiable configurations and directs attention to features such as grouping, participation rules, reward structure, outcome visibility, and task demands. The evidence does not establish any single competitive format as universally preferable or developmentally safer.

Support addresses the emotional, relational, and socio-moral demands that may arise during and after competitive episodes. Competition can involve frustration, coping, reconciliation, fairness judgments, self-interest, merit, equality, and group loyalty, extending its developmental significance beyond immediate task performance (Roseth et al., 2011; Pappert et al., 2017; Xiao et al., 2024a; Liu et al., 2025). Support therefore draws attention to children’s emotional participation, relational continuity, and interpretation of competitive outcomes. Broader teacher-focused research has identified strategies such as regulating competitive ambition, encouraging peer support, and promoting reflection among the approaches educators report using to help students cope with losing (Mateos-Moreno & Blanco-Ariza, 2026). However, the reviewed evidence does not establish which specific teacher-led emotional or moral scaffolding strategies are most effective.

The framework can therefore be summarized through three questions applicable when competition is used or emerges in an early childhood setting: How are children experiencing the competitive situation? Which features of the activity and its social organization may warrant adaptation? What emotional, relational, or socio-moral demands may require support? The framework is conceptual rather than prescriptive: Assess, Adapt, and Support were derived from recurring patterns across a heterogeneous evidence base, but their sequential or combined use has not been empirically validated. Research examining feasibility, acceptability, implementation conditions, and subsequent developmental outcomes is therefore required before the framework can be regarded as an evidence-based pedagogical protocol.

### 4.4. Implications for Research

The synthesis highlights several areas in which the evidence remains limited. Children’s perspectives are underrepresented, leaving relatively little knowledge of how young children themselves interpret competition, success, failure, fairness, and participation. Teacher mediation is similarly underexamined in ECE (M. Yıldız & Çiftçi, 2025), despite broader school-based evidence indicating that teachers actively modulate the salience of competition and social comparison in relation to their pedagogical goals and perceptions of individual learners (Goegan & Daniels, 2022). Longitudinal research is needed to distinguish immediate behavioral responses from longer-term developmental patterns, while direct cross-cultural designs are necessary to examine sociocultural mechanisms rather than infer them from studies conducted independently in different national contexts.

These gaps also define an empirical agenda for the Safe Zone Framework. The immediate priority is not effectiveness testing, but determining whether its constructs can be operationalized coherently and whether Assess, Adapt, and Support can be used feasibly and acceptably in authentic educational settings without categorizing or stigmatizing children. Subsequent implementation research could examine how these principles are enacted across classroom and sociocultural contexts, followed by controlled outcome studies evaluating their potential contribution to socio-emotional, socio-cognitive, socio-moral, and relational development.

### 4.5. Strengths and Limitations

The principal strength of this review lies in integrating evidence that has largely developed in separate studies. Socio-emotional, socio-cognitive, and socio-moral studies were synthesized together and subsequently interpreted across child/interactional, pedagogical/activity, and sociocultural contexts. This made it possible to identify cross-domain configurations that would remain less visible within outcome-specific reviews. Criterion-level MMAT appraisal also allowed methodological concerns to inform interpretation without treating heterogeneous research designs as inferentially equivalent.

These conclusions must nevertheless be interpreted within substantial evidentiary constraints. Competition was operationalized through highly heterogeneous tasks and measures, much of the quantitative evidence was cross-sectional or based on short experimental exposures, and representativeness and control of confounding varied considerably. Fixed-order designs, common-informant measurement, selective attrition, and measurement limitations further affected parts of the evidence base. The geographical distribution of studies was uneven, direct cross-cultural comparison was uncommon, and teacher mediation and children’s perspectives received relatively limited attention.

The review process introduces additional limitations. The restriction to English-language, peer-reviewed empirical journal articles published between 2000 and 2025 may have excluded relevant evidence, while disciplinary variation in terminology means that studies conceptualizing related phenomena under different labels may not have been retrieved. The birth-to-eight age range also encompasses substantial developmental variation. Although the review was conducted primarily by a single author and independent duplicate procedures were not implemented prospectively across all stages, a post hoc independent eligibility check was conducted for a randomly selected subset of 20 of the 80 full-text reports. This duplicate check focused on two central eligibility criteria—participant age and whether competition constituted a focal construct, condition, or experience—and yielded 95% agreement on overall eligibility decisions before resolution. Data extraction, MMAT appraisal, synthesis, and framework development remained author-led.

Most importantly, the Safe Zone Framework remains conceptual and provisional. The present synthesis supports its underlying questions more strongly than it supports any particular classroom procedure. Its feasibility, implementation, ethical implications, and developmental effects require direct empirical evaluation before it can be regarded as a validated pedagogical framework.

## 5. Conclusions

This systematic review indicates that competition in early childhood cannot be adequately understood as an inherently beneficial or harmful educational condition. Across the 56 included studies, reported developmental findings varied with children’s capacities and relational positioning, the demands and social architecture of competitive activities, and the sociocultural meanings through which competition was interpreted and socialized. The principal implication is therefore not that a particular form of competition should be universally promoted or avoided, but that competitive experiences need to be understood as context-dependent configurations rather than as a unitary pedagogical exposure.

The Safe Zone Framework translates this ecological interpretation into a preliminary evidence-informed conceptual structure for pedagogical reflection when competition is intentionally used or spontaneously emerges in early childhood settings. Its integrated use has not yet been empirically validated. Accordingly, the framework should presently be regarded as a conceptual proposal rather than an evidence-based classroom protocol. Classroom-based feasibility and implementation studies are needed to establish whether its principles can be applied consistently and acceptably, while longitudinal and cross-cultural research is required to examine their developmental and contextual relevance. Subsequent outcome studies should determine whether pedagogically structured use of the framework contributes to children’s socio-emotional, socio-cognitive, socio-moral, or relational outcomes. Until such evidence is available, its primary contribution lies in reframing competition from a binary pedagogical choice into an ecological question concerning how competitive experiences are structured, experienced, and supported.

## Figures and Tables

**Figure 1:**
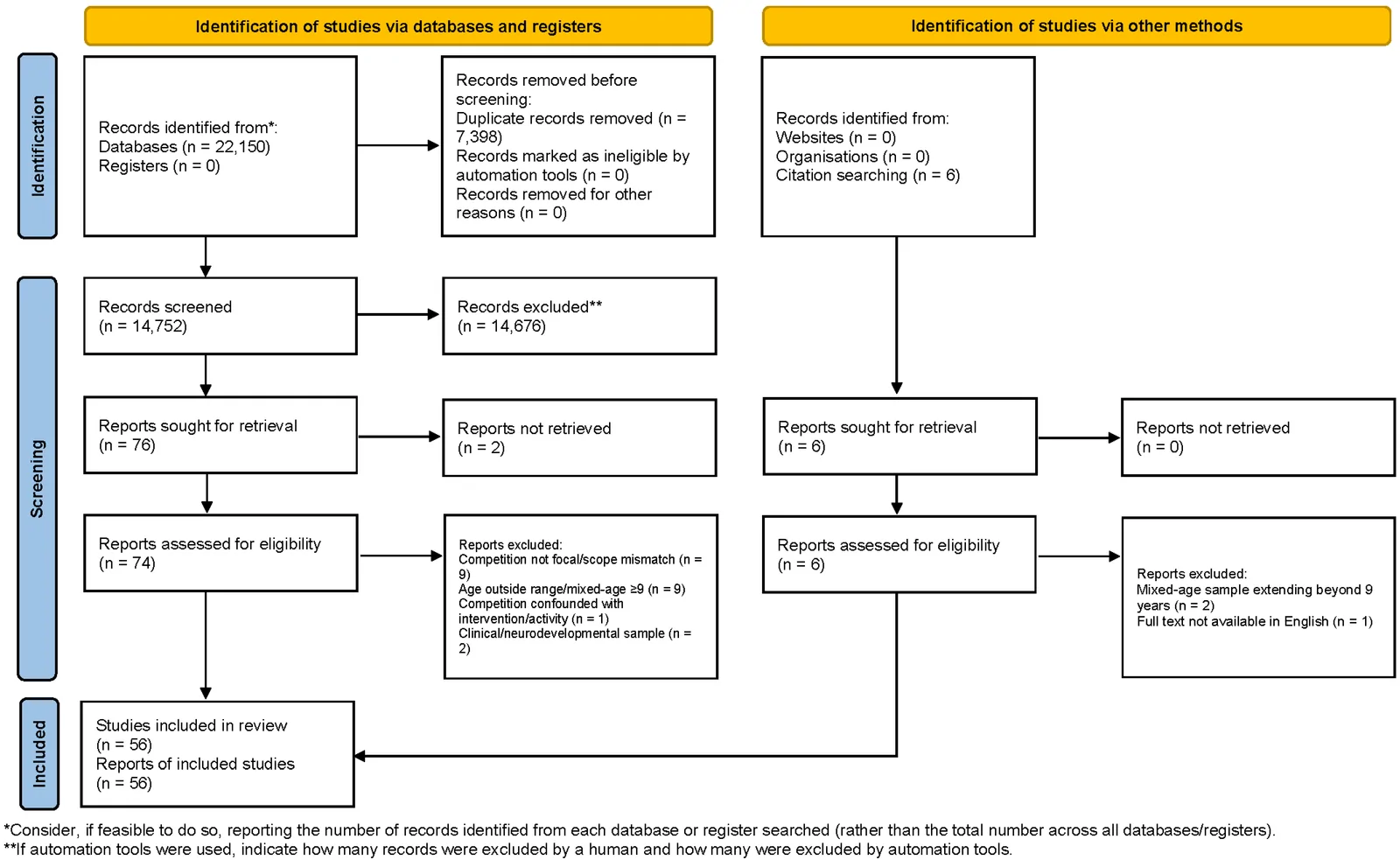
PRISMA 2020 flow diagram of study selection.

**Table 1:** Overview of studies.

Author(s)	Country/Context	Design	Participants	Primary Competition Construct/Form	Research Context/Setting	Competition Measures/Tasks	Main Findings
Donzella et al. (2000)	USA	Within-subject repeated-measures experimental study	*N* = 61 3–5 years	Competitive outcomes/experiences—loss within structured competition	Structured experimental/task context	Competitive Memory game	Losing increased negative affect and impulsivity; more surgent children showed greater stress reactivity to competition.
Benenson et al. (2001)	Canada	Experimental observational study	*N* = 72 71.33–84.28 months	Competitive situation/structure—interpersonal; dyadic vs. tetradic	Structured experimental/task context	Modified competitive board game	Competitive behavior was higher in tetrads than dyads, but this effect was driven primarily by boys. The emotional atmosphere, indexed by smiling, was more positive in dyads than tetrads.
Hughes et al. (2001)	UK	Longitudinal repeated-measures behavioral study	*N* = 40 + 40 = 80 5–7 years	Competitive outcomes/experiences—competitive loss	Structured experimental/task context	Snap! task	Hard-to-manage children showed more negative behavior during competitive loss than typically developing peers.
Paquette et al. (2003)	Canada	Correlational; six-month follow-up	*N* = 19 20–72 months	Competition orientation—motivation to win	Naturalistic interaction context	father–child rough-and-tumble play; non-aggressive competition measure	Motivation to win in father–child rough-and-tumble play was associated with greater non-aggressive competition.
Sheridan and Williams (2006)	Sweden	Qualitative/multimethod	*N* = 12 5 years	Competitive situation/structure—naturalistic peer competition	Qualitative/multimethod naturalistic context	Interviews, video observations, and children’s drawings	Constructive competition and cooperation frequently co-occurred in children’s shared activities.
Weinberger and Stein (2008)	USA	Cross-sectional comparative behavioral study	*N* = 64 5 years	Competitive situation/structure—interpersonal, resource-based	Structured experimental/task context	Bead collecting game	Boys showed more competitive behavior, while girls competed less primarily in same-gender groups.
Roseth et al. (2008)	USA	Short-term longitudinal naturalistic observational study	*N* = 91 29–60 months	Competitive situation/structure—naturalistic peer conflict	Naturalistic educational/peer context	Naturalistic conflict observation and PC–MC reconciliation coding	Teacher intervention was associated with a greater likelihood of separation following competitive conflict, but children who separated were also more likely subsequently to reconcile. Teacher intervention varied according to the form of aggression, indicating that adult mediation altered the natural conflict–peacemaking cycle rather than simply improving or worsening outcomes uniformly.
Ensor et al. (2011)	UK	Structured observational paradigm	*N* = 228 6 years	Competitive situation/structure—interpersonal, friend-dyad competition	Structured experimental/task context	Walk the Plank competitive board game	Aggression, disruption, arousal, and negative affect formed a comparable latent problem-behavior construct for boys and girls. Boys showed substantially higher problem-behavior scores than girls; individual differences also showed continuity with free-play behavior, especially among boys
Plusquellec et al. (2011)	Canada	Observational/correlational laboratory study	*N* ≈ 376 19 months	Competitive situation/structure—resource-based/resource control	Structured experimental/task context	Resource-control task	Higher pre-test cortisol was associated with lower resource control among boys, but not girls.
Roseth et al. (2011)	USA	Observational short-term longitudinal	*N* = 88; 29–60 months	Competitive situation/structure—naturalistic resource competition	Naturalistic educational/peer context	Naturalistic observations of coercive and prosocial resource competition	Coercive resource control decreased while prosocial resource-control strategies increased across the school year.
Guimond et al. (2012)	Canada	Longitudinal monozygotic twin-difference study	137 MZ twin pairs; mean age = 72 months	Competitive situation/structure—structured peer competition	Structured experimental/task context	Movie Viewer Situation	Parental overprotection predicted greater social reticence in competitive situations, particularly among boys.
Huyder and Nilsen (2012)	Canada	Within-subject dyadic behavioral study	*N* = 114 5–8 years	Competitive situation/structure—interpersonal	Structured experimental/task context	Puzzle task	Executive functioning did not predict behavior in the competitive context; better inhibitory control predicted fewer competitive behaviors only in the cooperative context.
Priewasser et al. (2013)	Austria	Correlational behavioral study	*N* = 71 3–5 years	Competitive situation/structure—strategic interpersonal competition	Structured experimental/task context	Bead-collecting game	False-belief understanding predicted more strategic competitive poaching moves.
Smith and LaFreniere (2013)	USA	Cross-sectional experimental behavioral study	*N* = 118; 4-, 6-, and 8-year-olds	Competitive situation/structure—strategic interpersonal competition	Structured experimental/task context	Competitive card-guessing/tactical-deception game	Strategic deception increased with age. Four-year-olds were largely non-strategic and rarely successful; 6-year-olds showed increasing strategic behavior and success, while 8-year-olds used more subtle deceptive strategies, achieved greater success, and showed greater understanding of the opponent’s expectations.
Strand et al. (2015)	USA	Cross-sectional comparative correlational study	*N* = 254 38–67 months	Competition orientation—competitive social values	Structured behavioral-assessment context	Social Orientation Choice Card	Competitive values predicted better adjustment among collectivist-background children but poorer adjustment among individualist-background children.
Tsiakara and Digelidis (2015)	Greece	Within-subject experimental study	*N* = 80 4–6 years	Competitive situation/structure—interpersonal	Structured experimental/task context	Build a Tower Task	Perceived performance and satisfaction were similar across the competitive and noncompetitive task contexts.
Zhu et al. (2015)	China	Non-randomized experimental study	*N* = 321 2–6 years	Competitive situation/structure—intergroup	Structured experimental/task context	Intergroup competition vs. noncompetition condition	Intergroup competition was associated with stronger ingroup prosocial sharing among younger than older children.
Goldvicht-Bacon and Diesendruck (2016)	Israel	Within-subject experimental study (Exp. 2)	*N* = 57; 56–83 months;	Competitive situation/structure—strategic interpersonal competition; culturally situated coordination	Structured experimental/task context	Competitive sticker-hiding coordination task with same- vs. different-culture partners	Five-year-olds adjusted their strategic choices according to the presumed cultural knowledge of their opponent: they selected cultural cues more often against different-culture than same-culture partners, indicating context-sensitive use of shared cultural knowledge in competition.
Schmidt et al. (2016)	Germany	Within-subject experimental study	*N* = 48 3–5 years	Competitive situation/structure—rule-governed interpersonal competition	Structured experimental/task context	Competitive game against a puppet varying rule adherence and game strategy	Five-year-olds, but not 3-year-olds, fully understood the dual normative structure of competitive games.
Jin et al. (2017)	China	Experimental study	Exp. 1 = 72 Exp. 2 = 24 Exp. 3 = 48 4 years	Competitive situation/structure—competition vs. cooperation	Structured experimental/task context	Fishing game	Cooperation, but not competition, improved children’s reasoning about others’ diverse desires.
Kalkman et al. (2017)	Norway	Qualitative ethnographic study	4-year-old preschool girls	Competitive situation/structure—naturalistic peer-status competition	Naturalistic educational/peer context	Naturalistic peer interactions focused on social competition for status	Social competition for peer position was intertwined with exclusion and relational aggression.
Mui et al. (2017)	China/ Netherlands	Experimental cross-cultural stud	55 Chinese and 31 Dutch 8-year-olds	Competitive outcomes/experiences—win/loss within structured competition	Structured experimental/task context	Competitive number-guessing game	Children’s smiles following winning and losing varied as a function of social and cultural context. Duchenne smiles occurred more frequently in paired play, and the effect of sociality on smile intensity was more pronounced among Chinese than Dutch children.
Pappert et al. (2017)	Canada	Randomized between-subject experimental study	*N* = 50 4–6 years	Competitive situation/structure—structured interpersonal competition	Structured experimental/task context	Coloring Task	Competition reduced prosocial resource allocation and generosity under some conditions.
Domberg et al. (2018)	Germany	Randomized between-subject experimental studies	*N* = 240 5–7 years	Competitive situation/structure—competition vs. cooperation	Structured experimental/task context	Zoo Game	Children produced fewer and more self-serving arguments in competitive than cooperative contexts, particularly at age 7.
Hu and Zhu (2018)	China	Experimental	*N* = 163 2–6 years	Competitive outcomes/experiences—win/loss and subsequent willingness to compete	Structured experimental/task context	Win/loss competition vs. noncompetition; willingness to compete	Younger children were more willing than older children to enter a subsequent competition.
Herrmann et al. (2019)	Germany	Experimental	Study 1 *N* = 128 Study 2 *N* = 64 5–8 years	Competitive situation/structure—competitive partner-choice	Structured experimental/task context	Competitive altruism dyadic sharing game	Eight-year-olds increased prosocial sharing when being observed by a peer who could later select them as a partner, indicating strategic reputation management in competitive partner-choice contexts. Five-year-olds did not show the same competitive-altruism effect.
P. Li et al. (2019)	China	Randomized experimental study	Exp 1 = 48 Exp 2 = 72 4–5 years	Competitive situation/structure—competition vs. cooperation	Structured experimental/task context	Fishing Game	Cooperation, but not competition, improved flexible visual perspective taking
Toppe et al. (2019)	Germany	Randomized experimental	*N* = 96 4–6 years	Competitive situation/structure—competition vs. cooperation	Structured experimental/task context	KoKo game	Children shared less with third parties after competitive than cooperative play; social inclusion was unaffected.
Castellano-Navarro et al. (2020)	Spain	Randomized experimental study	*N* = 48 6-year-olds	Competitive situation/structure—competition vs. cooperation; reward-allocation context	Structured experimental/task context	Competitive vs. cooperative reward-allocation context	Six-year-olds performed better in competition than cooperation on visual-perspective inference.
Roberts et al. (2020)	UK	Vignette-based quantitative study	*N* = 92 4–5 years	Competitive situation/structure—hypothetical resource competition	Structured behavioral-assessment context	Hypothetical resource-competition vignettes	Girls chose more prosocial and boys more coercive strategies, with responses also varying by opponent toughness.
Sai et al. (2020)	China	Cross-sectional correlational behavioral study	*N* = 97 3–4 years	Competitive situation/structure—strategic interpersonal competition	Structured experimental/task context	Competitive hide-and-seek game	Competitive-game lying was associated with aspects of mental-state understanding, particularly diverse-belief and belief-emotion understanding, whereas concealing-transgression lying showed no comparable associations.
Eriksson et al. (2021)	Sweden	Between-subject 6-week experimental	*N* = 65 4–5 years	Competitive situation/structure—competition vs. cooperation	Structured experimental/task context	Cooperative vs. competitive board games	Board-game type did not affect cooperative, prosocial, or antisocial behavior; cooperative games were enjoyed more.
Proft et al. (2021)	Germany	Randomized mixed-design experimental	*N* = 81 3 years	Competitive situation/structure—competitive goal relation	Structured experimental/task context	Competitive vs. incompatible-desire payoff condition	Three-year-olds could ascribe competitive desires but struggled to translate this understanding into strategic choices.
Reyes-Jaquez and Echols (2021)	USA	Randomized between-subject experimental	*N* = 88 3–4 years	Competitive situation/structure—competition vs. cooperation; reward structure	Structured experimental/task context	Candy finding game	Children endorsed partners’ claims more under cooperative than competitive reward rules; stronger EF supported rule-consistent responses, whereas ToM was unrelated.
Toppe et al. (2021)	Germany	Randomized between-dyad experimental	*N* = 144 4–6 years	Competitive situation/structure—competitive vs. cooperative vs. solitary play	Structured experimental/task context	Cooperative, competitive, or solitary game	Gaming context did not affect later prosociality, although competition increased physical engagement during play.
Tsoi et al. (2021)	USA	Between-subject experimental	*N* = 120 4 years	Competitive situation/structure—competition vs. cooperation	Structured experimental/task context	Stickers Task	Four-year-olds were better at inducing false beliefs in cooperation than competition.
M. Zhang and Sylva (2021)	UK	Sequential within-subject experimental	*N* = 68 3–4 and 6–7 years	Competitive situation/structure—intergroup	Structured experimental/task context	Noncompetitive followed by intergroup competitive context	Competition increased older children’s reliance on ingroup informants; younger children showed no clear shift.
Bayındır et al. (2022)	Türkiye	Cross-sectional correlational	*N* = 260 4–6 years	Competition orientation—task-oriented vs. other-referenced	Survey/questionnaire-based	PCQ	Better self-regulation predicted more task-oriented competition, while control skills predicted less other-referenced competition.
Emen Parlatan and Sığırtmaç (2022)	Türkiye	Case study	*N* = 14 48–66 months	Competitive outcomes/experiences—loss within structured competitive play	Structured competitive-play context	Structured competitive games	Competitive games elicited aggression after some losses but also prompted coping and problem-solving strategies.
Fish et al. (2023)	USA	Randomized between-subject experimental	*N* = 274 5–8 years	Competitive situation/structure—competition vs. collaboration vs. solo	Structured experimental/task context	Proportion-learning game under competition, collaboration, or solo conditions	Competition increased perseverance and task engagement, but older boys showed poorer proportional reasoning under competition.
Özçelik and Sapsağlam (2023)	Türkiye	Cross-sectional correlational	*N* = 287 4–6 years	Competition orientation—task-oriented vs. other-referenced	Survey/questionnaire-based context	PCQ	Task-oriented competition was positively related to social competence, whereas other-referenced competition was positively related to anger-aggression.
Prodan et al. (2023)	Romania	Non-randomized quantitative behavioral study	*N* = 75 6–8 years	Competitive situation/structure—strategic interpersonal competition	Structured experimental/task context	Computerized competitive hide-and-seek game	Children deceived familiar peers less than unfamiliar peers; ToM (Theory of Mind) and executive functions were associated with strategic deceptive performance.
Savage et al. (2023)	USA	Cross-sectional behavioral study	Final *N* = 211 43–66 months	Competition orientation—competitive social-value orientation	Structured behavioral-assessment context	Competitive-versus-cooperative resource-allocation choice	Competitive social values differed by family cultural background; English-speaking Latinx children were most likely to make competitive rather than cooperative choices. However, social-value orientation and its interaction with cultural background were unrelated to teacher-rated prosocial or problem behavior.
Uyanık et al. (2023)	Türkiye	Cross-sectional correlational	*N* = 202 4–6 years	Competition orientation—task-oriented vs. other-referenced	Survey/questionnaire-based context	PCQ	Play skills were positively related to task-oriented and negatively related to other-oriented competition.
Valcke and Nilsen (2023)	Canada	Within-subject repeated-measures experimental	*N* = 118 4–6 years	Competitive situation/structure—resource-based	Structured experimental/task context	Resource Distribution Task	Children shared more in cooperative contexts, while competition elicited more antagonistic verbal responses.
Xiao et al. (2023)	China	Between-subject experimental study	*N* = 374; 5–8 years	Competitive situation/structure—intergroup; win/loss; intergroup familiarity	Structured experimental/task context	Passing ball game	Competition outcome shaped merit-based resource allocation: children allocated more resources to their own group after winning and fewer after losing than in the noncompetitive condition. Intergroup familiarity reduced group bias primarily among younger children, with this effect weakening with age.
Grueneisen et al. (2024)	Germany	Two within-subject experimental studies	Exp. 1: *N* = 69; Exp. 2: *N* = 120; total *N* = 189; 4–7 years	Competitive situation/structure—competitive partner-choice	Structured experimental/task context	Cooperative vs. competitive partner-choice tasks	From about age 5, children selected task-relevant partners for cooperation but strategically avoided them as competitors.
S. Yıldız and Sak (2024)	Türkiye	Cross-sectional correlational survey study	*N* = 400 4–6 years	Competition orientation—task-oriented vs. other-referenced	Survey/questionnaire-based context	PCQ	Task-oriented competition was weakly negatively associated with generalized, separation, specific-phobia, and overall anxiety.
Xiao et al. (2024b)	China	Three experimental studies	Study 1: *N* = 208; Study 2: *N* = 280; Study 3: *N* = 250; ages 3–8 years	Competitive situation/structure—interpersonal/intergroup; secondary feature: win/loss	Structured experimental/task context	Passing ball game	Children were more merit-oriented after interpersonal wins but more equality-oriented after losses and intergroup competition.
Xiao et al. (2024a)	China	Between-subject experimental study	*N* = 310 5–6 and 7–8 year	Competitive situation/structure—intergroup; resource-allocation context	Structured experimental/task context	Finding football competition	Between-group competition shifted older children toward more equal allocations with ingroup collaborators, whereas younger children remained merit-oriented.
Çantaoğlu et al. (2025)	Türkiye	Cross-sectional relational survey study	*N* = 337 4–6 years	Competition orientation—other-oriented/dominance-related orientation	Survey/questionnaire-based context	PCQ	Other-oriented competition was weakly associated with digital-game addiction; dominance maintenance was the most prominent competition style.
Liu et al. (2025)	China	Qualitative multimethod study	*N* = 31 5–6 years	Competitive situation/structure—naturally occurring and child-conceptualized competition	Qualitative/multimethod naturalistic context	Child interviews, drawings, and observations focused on competition	Children viewed competition as involving comparison, winning, fairness, and striving; although winning was valued, friendship and participation were often considered more important.
Priewasser et al. (2025)	Austria/Germany	Within-subject experimental	*N* = 51 34–56 months	Competitive situation/structure—strategic competition/sabotage	Structured behavioral-assessment context	Cooperative and competitive sabotage-story scenarios	Children succeeded in cooperative scenarios but struggled with competitive sabotage; sabotage understanding was associated with false-belief understanding.
Xiao and Li (2025)	China	Experimental, two-study design; between-group competition	Study 1: *N* = 298; Study 2: *N* = 264; 3–8 years	Competitive situation/structure—intergroup; secondary feature: win/loss	Structured experimental/task context	Passing ball game	With increasing age, children made more equal allocations to in-group collaborators after winning but more generous allocations after losing. The findings suggest that children balance fairness concerns and in-group relational concerns differently according to competitive group status.
Q. Zhang et al. (2025)	China	Randomized between-subject experimental study	*N* = 120 4–5 years	Competitive situation/structure—competition vs. cooperation	Structured experimental/task context	Pony Crossing Game	Cooperative play increased sharing overall; girls showed more sharing than boys in the competitive condition.
Zhao et al. (2025)	China	Randomized between-subject experimental study	*N* = 601 3–8 years	Competitive situation/structure—intergroup; resource-allocation context	Structured experimental/task context	Building-Blocks Game	Competition increased own-side allocations; younger children prioritized self-interest, whereas older children increasingly favored their ingroup.

**Table 2:** Ecological Synthesis of Competition in Early Childhood.

Ecological Context	Key Dimensions	Cross-Study Synthesis	Evidence Boundaries
Child/ interactional	Developmental capacities; relational positioning; opponent behavior; strategic understanding; age/gender-linked variation	Competitive responses varied with children’s socio-cognitive and regulatory capacities, developmental level, and relational positioning. False-belief understanding and awareness of opponents’ intentions and expectations were associated with increasingly strategic competitive behavior, while regulatory capacities did not operate uniformly across contexts. Competitive behavior and problem responses also varied with age, gender, friendship relations, peer status, and opponents’ behavior.	Evidence derives from heterogeneous experimental, correlational, observational, and qualitative designs. Age-, gender-, or cognition-related differences should therefore be interpreted as context-sensitive patterns rather than stable indicators of children’s general “readiness” or disposition toward competition.
Pedagogical/ activity	Task demands; goal interdependence; interpersonal vs. intergroup structure; group size/composition; win–loss and reward structure; partner choice; adult mediation	Competitive activities differed in their performance, cognitive-strategic, distributive, and relational demands. Their developmental significance varied with the social architecture of the activity, including group size and composition, whether competition was interpersonal or intergroup, how winning and losing altered group status, whether future partner choice or reputation was salient, and how adults intervened in naturally occurring competitive conflict.	Direct comparisons across activity structures remain limited. Teacher intervention was examined in relatively few studies and did not show uniformly beneficial effects, while several experimental manipulations altered multiple features of the competitive situation simultaneously.
Sociocultural	Cultural background; culturally shared knowledge; family socialization; culturally situated meanings	The meaning and correlates of competition appeared to vary with sociocultural context. Competitive orientation was associated with behavioral adjustment differently across cultural-background groups, while culturally shared knowledge also shaped children’s strategic choices when opponents were framed as belonging to the same or a different cultural group. Qualitative and family-level evidence further suggested possible socialization pathways through sibling comparison, parenting practices, and competition-related narratives.	Sociocultural evidence remains comparatively limited and mixed. Some studies directly compared cultural groups, whereas others examined culturally framed interactions within a single cultural sample. Cultural differences in competitive choices or emotional expression should not be treated as evidence that culture consistently moderates competition–adjustment relations.

**Table 3:** Evidence-Informed Derivation of the Safe Zone Framework from the Review Synthesis.

Framework Component	Operational Meaning	Recurring Evidence Pattern	Contributing Studies (*n*)	Contradictory, Null, or Boundary Findings	MMAT-Informed Interpretation
Assess	Child-level variability/relational positioning	Age, self-regulation, emotional reactivity, social cognition, peer relations	Assess—*n* = 26: (Donzella et al., 2000; Hughes et al., 2001; Paquette et al., 2003; Ensor et al., 2011; Plusquellec et al., 2011; Huyder & Nilsen, 2012; Guimond et al., 2012; Priewasser et al., 2013; Smith & LaFreniere, 2013; Strand et al., 2015; Tsiakara & Digelidis, 2015; Goldvicht-Bacon & Diesendruck, 2016; Schmidt et al., 2016; Hu & Zhu, 2018; Roberts et al., 2020; Sai et al., 2020; Proft et al., 2021; Bayındır et al., 2022; Özçelik & Sapsağlam, 2023; Prodan et al., 2023; Savage et al., 2023; Uyanık et al., 2023; Grueneisen et al., 2024; S. Yıldız & Sak, 2024; Çantaoğlu et al., 2025; Priewasser et al., 2025)	Child-level capacities were not uniformly predictive across competitive contexts; some regulatory or socio-cognitive variables showed context-specific or null associations.	Supported across multiple designs, but several associations were derived from correlational or small-sample studies; child-level predictors were therefore interpreted as context-sensitive rather than deterministic.
Adapt	Competitive activity structure	Interpersonal/intergroup format, win/loss, resource structure, task demands	Adapt—*n* = 24: (Benenson et al., 2001; Sheridan & Williams, 2006; Weinberger & Stein, 2008; Zhu et al., 2015; Jin et al., 2017; Mui et al., 2017; Domberg et al., 2018; Herrmann et al., 2019; P. Li et al., 2019; Toppe et al., 2019; Castellano-Navarro et al., 2020; Eriksson et al., 2021; Reyes-Jaquez & Echols, 2021; Toppe et al., 2021; Tsoi et al., 2021; M. Zhang & Sylva 2021; Fish et al., 2023; Valcke & Nilsen, 2023; Xiao et al., 2023; Xiao et al., 2024a, 2024b; Xiao & Li, 2025; Q. Zhang et al., 2025; Zhao et al., 2025).	No single competitive format showed consistently beneficial or harmful effects; several task comparisons produced null or context-dependent findings.	Supported primarily by experimental and structured-task evidence, although incomplete reporting of randomization, blinding, fixed-order designs, and confounding in some studies limits causal certainty regarding specific competitive formats.
Support	Emotional, relational, socio-moral scaffolding	Coping after loss, fairness, peer relations, teacher mediation	Support—*n* = 7: (Roseth et al., 2008; Roseth et al., 2011; Kalkman et al., 2017; Pappert et al., 2017; Emen Parlatan & Sığırtmaç, 2022; Xiao et al., 2024a; Liu et al., 2025)	Direct evidence on teacher-led support was limited, and the review did not establish which emotional, relational, or moral scaffolding strategies are most effective.	Supported by converging emotional, relational, and socio-moral findings, but direct evidence on teacher-mediated support was sparse and often based on observational, qualitative, or context-specific studies; pedagogical implications were therefore treated as provisional.

Note. All 56 included studies contributed to at least one framework component. Studies could contribute to more than one component when their findings informed more than one recurring pattern; therefore, component counts are not mutually exclusive and do not sum to 56.

## Data Availability

All data supporting the findings of this review are contained within the article, Appendix A, and the Supplementary Materials. No new primary datasets were generated for this review.

## Supplementary Materials

The following supporting information can be downloaded at: https://www.mdpi.com/article/10.3390/bs16101746/s1, Table S1: Database-Specific Search Strategies; Table S2: Reports Excluded Following Full-Text Assessment and Reasons for Exclusion; Table S3: Study-Level Results and Methodological Interpretation of Included Quantitative Studies. Refs. (Baurain & Nader-Grosbois, 2012; Carlo et al., 2001; Crewdson et al., 2023; Etel & Slaughter, 2019; Fischer et al., 2018; Hamamouche & Cordes, 2023; Hou et al., 2023; Houser & Schunk, 2009; Karaarslan et al., 2025; Karlsson et al., 2017; Lawrence, 2018; McMahon et al., 2021; Rhodes & Brickman, 2011; Roy & Benenson, 2002; Wei & Hendrix, 2009; Yu et al., 2024; X. Zhang et al., 2023; Eastin, 2007; Kajanus, 2019; H. Li et al., 2020; Lobel et al., 2019; McGuire et al., 2019; Yarmolovsky & Geva, 2024; Zhou et al., 2025) are cited in the supplementary materials.

## Abbreviations

The following abbreviations are used in this manuscript:

ToM	Theory of Mind
ECE	Early childhood education
MMAT	Mixed Methods Appraisal Tool

## Appendix A

**Table A1:** Methodological Quality Appraisal of Included Studies Using the MMAT.

Study	S1	S2	C1	C2	C3	C4	C5	Key Methodological Concern
Qualitative studies—MMAT Category 1								
Sheridan and Williams (2006)	Y	Y	Y	Y	Y	Y	Y	Small, context-specific sample; transferability is limited despite triangulation across interviews, observations, and drawings.
Kalkman et al. (2017)	Y	Y	Y	Y	Y	Y	Y	Interpretation of social competition relies primarily on an in-depth analysis of a single focal video episode.
Emen Parlatan and Sığırtmaç (2022)	Y	Y	Y	Y	Y	Y	Y	Small single-class sample limits transferability, although video data were independently coded and disagreements resolved by consensus.
Liu et al. (2025)	Y	Y	Y	Y	Y	Y	Y	Single-kindergarten sample and brief interviews constrain transferability despite multimethod data collection.
Randomized quantitative studies—MMAT Category 2								
Jin et al. (2017)	Y	Y	CT	Y	Y	CT	Y	Random assignment reported, but sequence generation/concealment and assessor blinding insufficiently described.
Mui et al. (2017)	Y	Y	Y	CT	Y	Y	Y	Social condition was randomized and facial expressions were coded by a condition-blinded FACS coder, but cultural group membership was naturally occurring and baseline equivalence across conditions/cultural groups was not established.
Pappert et al. (2017)	Y	Y	CT	Y	Y	CT	Y	Randomization method and outcome-assessor blinding not reported in sufficient detail.
Domberg et al. (2018)	Y	Y	CT	Y	Y	CT	Y	Random assignment reported, but generation/concealment and assessor blinding unclear.
Herrmann et al. (2019)	Y	Y	Y	CT	N	CT	Y	Random assignment was reported, but substantial post-assignment exclusions—particularly in Study 2—and incompletely documented outcome-assessor blinding warrant caution.
P. Li et al. (2019)	Y	Y	CT	Y	Y	CT	Y	Post-allocation exclusions transparent; randomization procedure and assessor blinding incompletely reported.
Toppe et al. (2019)	Y	Y	CT	Y	CT	N	Y	Additional dyad exclusions and only partial outcome-assessor blinding.
Castellano-Navarro et al. (2020)	Y	Y	CT	Y	Y	Y	Y	Strong blinded coding, but randomization sequence/concealment not described.
Eriksson et al. (2021)	Y	Y	CT	Y	CT	N	N	Variable intervention exposure/task completion and very low reliability for competitive-behavior coding.
Proft et al. (2021)	Y	Y	CT	CT	Y	CT	CT	Randomization procedure, baseline equivalence, assessor blinding, and adherence incompletely documented.
Reyes-Jaquez and Echols (2021)	Y	Y	CT	Y	Y	N	Y	Experimenter administered the condition and recorded behavioral responses; assessor was not blinded.
Toppe et al. (2021)	Y	Y	CT	Y	Y	CT	Y	Preregistered observer baseline excluded from inferential analyses; condition blinding not explicit.
Fish et al. (2023)	Y	Y	CT	Y	Y	CT	Y	Stratified random assignment reported, but sequence/concealment and assessor blinding insufficiently detailed.
Q. Zhang et al. (2025)	Y	Y	CT	CT	Y	CT	Y	Baseline comparability and assessor blinding incompletely reported.
Zhao et al. (2025)	Y	Y	CT	Y	Y	CT	Y	Large balanced sample, but randomization procedure and condition-blinding not fully reported.
Non-randomized quantitative studies—MMAT Category 3								
Donzella et al. (2000)	Y	Y	N	Y	Y	N	Y	University laboratory-school sample; fixed Win–Lose–Win sequence confounds condition with order/time.
Benenson et al. (2001)	Y	Y	N	Y	Y	Y	Y	Consent-based three-school sample limits representativeness; however, target children were randomly selected within tetrads and dyad–tetrad order was counterbalanced.
Hughes et al. (2001)	Y	Y	N	Y	Y	Y	Y	Extreme-group sampling and longitudinal attrition; friendship partners changed more often in the hard-to-manage group.
Paquette et al. (2003)	Y	Y	N	N	N	N	Y	Competition association based on only n = 19; low reliability and limited confounder adjustment.
Roseth et al. (2008)	Y	Y	N	Y	Y	N	Y	Single laboratory-preschool sample; naturally occurring teacher intervention may be confounded with conflict severity and contextual characteristics.
Weinberger and Stein (2008)	Y	Y	N	Y	Y	N	Y	Small convenience sample; same- versus mixed-gender grouping was not randomized.
Ensor et al. (2011)	Y	Y	N	Y	CT	N	Y	Longitudinal cohort and attrition constrain representativeness and outcome completeness; observational friend-dyad comparisons do not fully control contextual confounding.
Plusquellec et al. (2011)	Y	Y	N	Y	N	N	Y	Twin subsample, substantial missingness, and pre-test cortisol potentially affected by laboratory novelty.
Roseth et al. (2011)	Y	Y	N	Y	Y	CT	Y	Single laboratory-school setting and residual confounding in naturally occurring resource-control relationships.
Guimond et al. (2012)	Y	Y	Y	Y	N	Y	Y	Strong genetically informed design, but complete data were available for only about three-quarters of pairs.
Huyder and Nilsen (2012)	Y	Y	N	Y	Y	N	Y	Cooperative context always preceded competitive context, creating order/familiarity confounding.
Priewasser et al. (2013)	Y	Y	N	Y	Y	Y	Y	Local volunteer sample; some exclusions based on unusual game behavior.
Smith and LaFreniere (2013)	Y	Y	N	Y	Y	N	Y	Convenience sample drawn predominantly from White, middle- and working-class families in small Maine communities; age-group comparisons did not adjust for broader developmental or sociodemographic confounding, although behavioral coding showed very high inter-rater reliability.
Strand et al. (2015)	Y	Y	N	Y	Y	Y	Y	Restricted low-income Head Start sample limits population generalizability.
Tsiakara and Digelidis (2015)	Y	Y	N	CT	Y	N	Y	Measure reliability/validity insufficiently reported; competitive comparison also altered peer presence and comparison target.
Zhu et al. (2015)	Y	Y	N	Y	Y	N	Y	Competition/control condition not randomized and controls recruited from only three of five preschools.
Goldvicht-Bacon and Diesendruck (2016)	Y	Y	N	Y	Y	Y	Y	Small geographically and culturally homogeneous sample drawn from two kindergartens; counterbalancing reduced order-related confounding, but the culturally framed partner manipulation and hypothetical interaction constrain population and ecological generalizability.
Schmidt et al. (2016)	Y	Y	N	Y	Y	Y	Y	Small local sample; otherwise strong counterbalancing and behavioral coding.
Hu and Zhu (2018)	Y	Y	N	Y	Y	N	Y	Win/loss reflected actual performance and was therefore confounded with ability/effort.
Roberts et al. (2020)	Y	Y	N	CT	Y	CT	Y	Local sample; adapted hypothetical vignette measure lacked reported reliability/coder agreement.
Sai et al. (2020)	Y	Y	N	Y	Y	Y	Y	Two-preschool sample; correlational design prevents causal interpretation.
Tsoi et al. (2021)	Y	Y	N	Y	Y	CT	Y	Convenience recruitment and unclear allocation procedure; alternative explanations were only partly controlled.
M. Zhang and Sylva (2021)	Y	Y	CT	Y	Y	N	Y	Noncompetitive condition always preceded competition; carryover/order effects cannot be separated.
Bayındır et al. (2022)	Y	Y	CT	Y	Y	Y	Y	Same teachers rated regulation and competition, creating common-rater bias.
Özçelik and Sapsağlam (2023)	Y	Y	N	Y	Y	N	Y	Nonprobability sampling and primarily bivariate analyses without adjusted confounder models.
Prodan et al. (2023)	Y	Y	N	Y	Y	Y	Y	Single urban, middle-to-high-income school sample limits representativeness; the novel computerized deception task and partially unbalanced familiarity manipulation constrain generalizability and the scope of familiarity comparisons.
Savage et al. (2023)	Y	Y	N	Y	N	N	Y	Restricted Head Start sample and comprehension-based exclusions reduced the analytic sample; cultural-background comparisons remain observational and susceptible to residual confounding.
Uyanık et al. (2023)	Y	Y	N	Y	Y	N	Y	Convenience sample and unadjusted bivariate analyses.
Valcke and Nilsen (2023)	Y	Y	N	Y	Y	Y	Y	Local consent-based sample; fictional partners improve control but reduce ecological authenticity.
Xiao et al. (2023)	Y	Y	N	Y	Y	N	Y	Single-region Han Chinese sample limits representativeness; competitive win/loss was performance-determined and familiarity conditions incorporated naturally occurring friendship relations, leaving potential residual confounding.
Grueneisen et al. (2024)	Y	Y	N	Y	Y	Y	Y	Participant-database/local recruitment and hypothetical partner-choice tasks constrain generalizability.
S. Yıldız and Sak (2024)	Y	Y	Y	Y	Y	N	Y	Probability sampling was strong, but relationships were based on bivariate correlations without multivariable confounder adjustment.
Xiao et al. (2024b)	Y	Y	N	Y	Y	N	Y	Win/loss was performance-determined; interpersonal/intergroup comparisons also relied on separate samples.
Xiao et al. (2024a)	Y	Y	N	Y	Y	N	Y	Noncompetition and merit were randomized, but actual competitive win/loss remained performance-determined.
Çantaoğlu et al. (2025)	Y	Y	N	CT	Y	N	Y	Purposive sample; current-sample reliability insufficiently reported for one principal measure; analyses unadjusted.
Priewasser et al. (2025)	Y	Y	N	Y	N	Y	Y	18/69 children excluded, including comprehension/familiarization failures, creating potentially selective attrition.
Xiao and Li (2025)	Y	Y	N	Y	Y	N	Y	Although some condition elements were randomized, competitive win/loss depended on actual performance; consequently, competition outcome remained potentially confounded with performance-related characteristics.

Note. Y = Yes; N = No; CT = Can’t tell. S1 and S2 refer to the two MMAT screening questions. C1–C5 correspond to the five design-specific criteria; criteria 1.1–1.5 apply to qualitative studies, 2.1–2.5 to randomized quantitative studies, and 3.1–3.5 to non-randomized quantitative studies. An outcome-completeness threshold of ≥80% was used as a consistent interpretive criterion where participant retention or missing outcome data could be determined. Criterion-level judgments were retained rather than combined into an overall quality score.
