# Supplementary material for: Competition in Early Childhood: A Systematic Review of Developmental Evidence and a Preliminary Ecological Framework
Source: Behav Sci (Basel). 2026 Sep 24;16(10):1746. doi: 10.3390/bs16101746 (PMC13701259; doi:10.3390/bs16101746)
Supplement: Supplementary file 1 [file behavsci-16-01746-s001.zip › behavsci-4537045-supplementary.pdf]

Supplementary Table S1. Database-Specific Search Strategies

Information source	Platform / interface	Field selection	Complete search syntax	Limits / filters / subject exclusions	Last searched	Records retrieved
**Web of Science**	Web of Science Core Collection	TS= (Topic)	((competition OR competitive OR rivalry OR rival* OR "competitive play" OR "competitive game*" OR "social comparison" OR rank* OR "zero-sum" OR winning OR losing OR win OR "peer status" OR "resource competition") AND (social OR emotion* OR aggression OR motivation OR "self-regulation" OR prosocial* OR cooperation OR fairness OR "resource allocation" OR "moral reasoning" OR "theory of mind" OR ToM OR deception OR sharing OR peer*) AND ("early childhood" OR child* OR preschool* OR kindergarten* OR toddler* OR "young child*" OR "primary school"))	Document type: Article; Language: English; subject areas outside review scope excluded, including zoology, sport sciences, cell biology, pharmacology, surgery, endocrinology, radiology, molecular biology, cardiac and cardiovascular sciences, urology, and virology	16 Sep 2026	9,127
**Scopus**	Scopus	TITLE-ABS-KEY	(competition OR competitive OR rivalry OR rival* OR "competitive play" OR "competitive game*" OR "social comparison" OR rank* OR "zero-sum" OR winning OR losing OR win OR "peer status" OR "resource competition") AND (social OR emotion* OR aggression OR motivation OR "self-regulation" OR prosocial* OR cooperation OR fairness OR "resource allocation" OR "moral reasoning" OR "theory of mind" OR ToM OR deception OR sharing OR peer*) AND ("early childhood" OR child* OR preschool* OR kindergarten* OR toddler* OR "young child*" OR "primary school")	Document type: Article; Source type: Journal; Language: English; subject areas excluded: Biochemistry, Genetics and Molecular Biology; Immunology and Microbiology; Pharmacology, Toxicology and Pharmaceutics; Veterinary; Energy; Chemistry; Physics and Astronomy; Chemical Engineering	16 Sep 2026	8,171
**ERIC**	ERIC	ERIC indexed search	(competition OR competitive OR rivalry OR rival* OR "competitive play" OR "competitive game*" OR "social comparison" OR rank* OR "zero-sum" OR winning OR losing OR win OR "peer status" OR "resource competition") AND (social OR emotion* OR aggression OR motivation OR "self-regulation" OR prosocial* OR cooperation OR fairness OR "resource allocation" OR "moral reasoning" OR "theory of mind" OR ToM OR deception OR sharing OR peer*) AND ("early childhood" OR child* OR preschool* OR kindergarten* OR toddler* OR "young child*" OR "primary school")	pubyearmin:2000; pubyearmax:2025; Peer-reviewed only	16 Sep 2026	800

**PubMed**	PubMed	Title / Abstract	(competition OR competitive OR rivalry OR rival* OR "competitive play" OR "competitive game*" OR "social comparison" OR rank* OR "zero-sum" OR winning OR losing OR win OR "peer status" OR "resource competition") AND (social OR emotion* OR aggression OR motivation OR "self-regulation" OR prosocial* OR cooperation OR fairness OR "resource allocation" OR "moral reasoning" OR "theory of mind" OR ToM OR deception OR sharing OR peer*) AND ("early childhood" OR child* OR preschool* OR kindergarten* OR toddler* OR "young child*" OR "primary school")	Age filters: Newborn: birth–1 month; Infant: birth–23 months / 1–23 months; Preschool Child: 2–5 years; Child: 6–12 years; Language: English	16 Sep 2026	1,860
**PsycINFO**	**APA PsycINFO / EBSCO-host**	XB / Title-Abstract	(competition OR competitive OR rivalry OR rival* OR "competitive play" OR "competitive game*" OR "social comparison" OR rank* OR "zero-sum" OR winning OR losing OR win OR "peer status" OR "resource competition") AND (social OR emotion* OR aggression OR motivation OR "self-regulation" OR prosocial* OR cooperation OR fairness OR "resource allocation" OR "moral reasoning" OR "theory of mind" OR ToM OR deception OR sharing OR peer*) AND ("early childhood" OR child* OR preschool* OR kindergarten* OR toddler* OR "young child*" OR "primary school")	Source type: Article	16 Sep 2026	2,192
				**Total before deduplication**		22,150

Supplementary Table S2. Reports Excluded Following Full-Text Assessment and Reasons for Exclusion

No.	Study	Reason for exclusion
1	Lawrence (2018)	Competition emerged incidentally within peer digital-play interactions rather than constituting a predefined focal variable or condition.
2	McMahon et al. (2021)	Competition served as the task context rather than the focal construct; the primary outcome concerned children's use of kinematic cues for action prediction.
3	Hou et al. (2023)	Competition was not examined as a focal construct or condition; the study primarily addressed social competence and Theory of Mind.
4	Wei & Hendrix (2009)	Competition was embedded in an educational game context, whereas the principal outcome concerned recall of game content rather than the predefined developmental domains.
5	Rhodes & Brickman (2011)	Competition was presented as information about third-party social groups rather than as a competitive experience involving participating children; the primary focus was children's representations of novel social categories.
6	Etel & Slaughter (2019)	Competition was not examined as a focal construct or condition; the study investigated Theory of Mind and peer cooperation across joint and independent play contexts.
7	X. Zhang et al. (2023)	Competition was not a focal construct or outcome; the study primarily examined other-benefiting lying and its associations with Theory of Mind and empathy.
8	Karlsson et al. (2017)	Competition was not examined as a focal variable or condition; the study focused on children's negotiation of rules, cheating, justice, fairness, and moral orders during naturally occurring board-game interactions.
9	Houser & Schunk (2009)	Participants were outside the predefined birth-to-8-year age range; the study focused on children aged 8–10 years.
10	Roy & Benenson (2002)	The participant sample extended beyond the predefined birth-to-8-year age range.
11	Fischer et al. (2018)	The mixed-age sample extended beyond the predefined birth-to-8-year age range and was excluded under the age eligibility criterion.
12	Carlo et al. (2001)	The mixed-age sample extended beyond the predefined birth-to-8-year age range (37–140 months).
13	Hamamouche & Cordes (2023)	The mixed-age sample extended beyond the predefined birth-to-8-year age range (3.15–9.92 years).
14	Crewdson et al. (2023)	Competition was confounded with intervention and activity structure; Tootling and interdependent group rewards differed across the comparison conditions.
15	Yu et al. (2024)	The study centered on a diagnosed neurodevelopmental population, comparing autistic preschoolers with typically developing peers; studies focused on clinical/neurodevelopmental samples were excluded.
16	Baurain & Nader-Grosbois (2012)	Clinical/neurodevelopmental sample: the study compared children with intellectual disability with typically developing children
17	McGuire et al. (2019)	The participant sample extended beyond the predefined birth-through-8-years age range and included adolescents and adults.
18	Yarmolovsky & Geva (2024)	The participant sample extended beyond the predefined birth-through-8-years age range; children were 8–12 years old.
19	Kajanus (2019)	The participant sample included children aged 9 years and therefore extended beyond the predefined birth-through-8-years age range.
20	H. Li et al. (2020)	The study examined competitive movement and evacuation dynamics rather than socio-emotional, socio-cognitive, or socio-moral developmental outcomes relevant to the review.

No.	Study	Reason for exclusion
21	Zhou et al. (2025)	The participant sample was outside the predefined birth-through-8-years age range (M age = 9.85 years).
22	Karaarslan et al. (2025)	Full text not available in English
23	Lobel et al. (2019)	The participant sample extended beyond the predefined birth-through-8-years age range (8.31–12.68 years).
24	Eastin (2007)	The participant sample was entirely outside the predefined birth-through-8-years age range; participants were 18–41 years old.

Supplementary Table S3. Study-Level Results and Methodological Interpretation of Included Quantitative Studies

Study	N / Design	Outcome / comparison relevant to the review	Effect direction / reported finding	Key statistical result as reported	Uncertainty / precision	MMAT-informed interpretive note
Donzella et al. (2000)	N = 61 3–5 years; Within-subject repeated-measures experimental study	Competitive outcomes/experiences — loss within structured competition; Competitive Memory game	Losing increased negative affect and impulsivity; more surgent children showed greater stress reactivity to competition.	Game-period effects: positive affect, $F(3,116)=28.7$, $p<.001$; sad affect, $F(3,116)=24.6$, $p<.01$; tense/angry affect, $F(3,116)=10.8$, $p<.01$. Impulsivity differed across periods, $F(2,116)=4.97$, $p<.01$, with Lose M=.75, SD=.60 vs Win I M=.34, SD=.61. Surgency correlated with tense/angry affect during loss, $r(53)=.29$, $p<.05$. High surgency was associated with cortisol response, Fisher's exact $p=.02$.	Means/SDs and correlations reported; CIs and standardized effects NR.	Interpret with the following criterion-level MMAT concern in mind: University laboratory-school sample; fixed Win–Lose–Win sequence confounds condition with order/time.
Benenson et al. (2001)	N = 144 participants; 72 target children analyzed; ages approximately 6–7 years; within-subject dyad–tetrad comparison with counterbalanced order	Competitive situation/structure — interpersonal; dyadic vs. tetradic; Modified competitive board game	Competitive behavior was higher in tetrads than dyads, but this effect was driven primarily by boys. The emotional atmosphere, indexed by smiling, was more positive in dyads than tetrads.	Group-size effect on competitive moves: $F(1,33)=10.17$, **$p<.005$**; tetrads M=44.00, SD=35.26, dyads M=28.48, SD=31.99. Group size × gender: $F(1,33)=4.02$, $p<.05$. Smiling: group-size effect $F(1,28)=11.06$, $p=.002$; dyads M=4.11, SD=.80, tetrads M=3.53, SD=.88.	Means/SDs reported; CI/effect size NR.	MMAT-informed caution: Three-school consent sample; dyad/tetrad order was counterbalanced, but the sampling frame and naturally occurring gender composition limit representativeness.
Hughes et al. (2001)	N = 40 + 40 = 80 5-7 years; Longitudinal repeated-measures behavioral study	Competitive outcomes/experiences — competitive loss; Snap! task	Hard-to-manage children showed more negative behavior during competitive loss	Overall repeated-measures group effect: $F(1,56)=7.14$, $p<.01$. Age 5: hard-to-manage M=7.08, SD=5.48 vs control M=3.60, SD=3.76, $F(1,76)=9.92$,	Means/SDs and regression coefficients reported; CI/effect size NR.	Interpret with the following criterion-level MMAT concern in mind: Extreme-group sampling and longitudinal attrition; friendship partners changed

Study	N / Design	Outcome / comparison relevant to the review	Effect direction / reported finding	Key statistical result as reported	Uncertainty / precision	MMAT-informed interpretive note
			than typically developing peers.	p=.002**.** Age 7: M=9.00, SD=6.02 **vs** M=6.46, SD=5.06**,** F(1,63)=3.82, **p=.055**. In the hard-to-manage group, violent pretend play predicted later negative behavior (age 5 β=.37, age 7 β=.54**,** both *p*<.05).		more often in the hard-to-manage group.
Paquette et al. (2003)	N = 19 20–72 months; Correlational; six-month follow-up	Competition orientation — motivation to win; father–child rough-and-tumble play; non-aggressive competition measure	Motivation to win in father–child rough-and-tumble play was associated with greater non-aggressive competition.	Motivation to win × non-aggressive competition: ϱ=.69, p<.01, n=19**.** RTP duration × non-aggressive competition: ϱ=.40, p<.10, n=19**.**	Correlations and *p* values reported; CI NR. Small n and low internal consistency of the non-aggressive competition measure.	Interpret with the following criterion-level MMAT concern in mind: Competition association based on only n=19; low reliability and limited confounder adjustment.
Weinberger and Stein, (2008)	N =64 5 years; Cross-sectional comparative behavioral study	Competitive situation/structure — interpersonal, resource-based; Bead collecting game	Boys showed more competitive behavior, while girls competed less primarily in same-gender groups.	Gender: F(1,60)=36.90, p<.05**;** gender composition: F(1,60)=19.80, p<.05**;** gender × composition**:** F(1,60)=31.58, p<.05**.** Competitive moves: boys M=.83, SD=.23**,** girls M=.44, SD=.43**;** girls same-gender M=.12, SD=.27 vs mixed-gender M=.76, SD=.30**.** Strategic moves: gender F(1,47)=.177, p>.05**,** composition F(1,47)=.001, p>.05**,** interaction F(1,47)=.004, p>.05**.**	Means/SDs reported; exact significant *p* values beyond *p*<.05 and effect sizes/CIs NR.	Interpret with the following criterion-level MMAT concern in mind: Small convenience sample; same- versus mixed-gender grouping was not randomized.

Study	N / Design	Outcome / comparison relevant to the review	Effect direction / reported finding	Key statistical result as reported	Uncertainty / precision	MMAT-informed interpretive note
Roseth et al. (2008)	N = 91 29–60 months; Short-term longitudinal naturalistic observational study	Competitive situation/structure — naturalistic peer conflict;	Teacher intervention was associated with a greater likelihood of separation following competitive conflict, but children who separated were also more likely subsequently to reconcile. Teacher intervention varied according to the form of aggression, indicating that adult mediation altered the natural conflict–peacemaking cycle rather than simply improving or worsening outcomes uniformly.	Of 139 conflicts with teacher intervention, 92 (66%) ended in separation and 47 (34%) in together outcomes, exact binomial $p<.01$; individual-level comparison $z=-3.00$, $p<.01$. With intervention, PC–MC analysis: 35 attracted, 11 dispersed, 26 neutral pairs, exact binomial $p<.01$; dyadic $z=-3.61$, $p<.01$; CCT=33%. Without intervention: 7 attracted, 5 dispersed, 9 neutral, exact binomial $p=.77$; dyadic $z=-.57$, $p=.56$; CCT=9%. ǀ Frequencies, percentages, exact binomial tests, z statistics, and corrected conciliatory tendency reported; CIs/effect sizes NR.	Naturalistic design; teacher intervention was not randomized and may reflect conflict severity/context despite strong observer reliability.	MMAT-informed caution: Single university laboratory preschool; teacher intervention was naturally occurring and may be confounded with conflict severity/context despite strong observer reliability.
Ensor et al. (2011)	N =228 6-years; Structured observational paradigm	Competitive situation/structure — interpersonal, friend-dyad competition; Walk-the-Plank competitive board-game	Aggression, disruption, arousal, and negative affect formed a comparable latent problem-behavior construct for boys and girls. Boys showed substantially higher problem-behavior	Standardized latent problem-behavior mean was .60 higher for boys, $z=3.78$, $p<.01$. Observed indicators: aggression boys $M=.58$, $SD=$**.85**, girls $M=.21$, $SD=.48$; disruption 2.07 (.92) vs 1.81 (.71); arousal 3.40 (.94) vs 3.13 (.89); negativity 2.25 (.99) vs 1.81 (.99). Associa-	Standardized estimates and SDs reported; CIs NR.	MMAT-informed caution: Low-income longitudinal cohort with attrition from the original consented sample; observational friend-dyad data limit causal interpretation.

Study	N / Design	Outcome / comparison relevant to the review	Effect direction / reported finding	Key statistical result as reported	Uncertainty / precision	MMAT-informed interpretive note
			scores than girls; individual differences also showed continuity with free-play behavior, especially among boys	tion with teacher-rated total difficulties: boys .31, z=2.67, p<.01, girls .05, z=.41, p=.68; difference z=1.98, p<.05.		
Plusquellec et al. (2011)	N = 376 toddlers; age 19 months; observational/correlational laboratory study	Pre-test cortisol and competitive resource control (boys)	Higher pre-test cortisol was associated with less resource control in the competitive task among boys, but not girls.	r = −0.17, p = .02; girls: r = .09, p = .21	CI/SE NR; p values reported.	Interpret with the following criterion-level MMAT concern in mind: Twin subsample, substantial missingness, and pre-test cortisol potentially affected by laboratory novelty.
Roseth et al. (2011)	N = 88; 29–60 months; Observational short-term longitudinal	Competitive situation/structure — naturalistic resource competition; Naturalistic observations of coercive and prosocial resource competition	Coercive resource control decreased while prosocial resource-control strategies increased across the school year.	Coercive linear trend: β = −.10, SE=.02, 95% CI [−.15, −.06], z=−4.50, p<.001. Prosocial linear trend: β=.08, SE=.01, 95% CI [.05, .12], z=4.90, p<.001. Controlling for age, dominance predicted coercive control: β=.18, SE=.06, 95% CI [.05, .31], z=2.76, p<.01, and prosocial control: β=.14, SE=.07, 95% CI [.01, .29], z=1.95, p<.05.	SEs and 95% CIs reported.	Interpret with the following criterion-level MMAT concern in mind: Single laboratory-school setting and residual confounding in naturally occurring resource-control relationships.
Guimond et al. (2012)	137 MZ twin pairs; mean age = 72 months; Longitudinal monozygotic twin-difference study	Competitive situation/structure — structured peer competition; Movie Viewer Situation	Parental overprotection predicted greater social reticence in competitive situations, particularly among boys.	Boys: maternal overprotection β=.31, SE=.15, p=.05; paternal overprotection β=.38, SE=.14, p=.02. Girls: paternal hostility × paternal depressive symptoms β=.42, SE=.12, p=.01. Sex-group	Standardized βs and SEs reported; CIs NR.	Interpret with the following criterion-level MMAT concern in mind: Strong genetically informed design, but complete data were available for only about three-quarters of pairs.

Study	N / Design	Outcome / comparison relevant to the review	Effect direction / reported finding	Key statistical result as reported	Uncertainty / precision	MMAT-informed interpretive note
				contrasts: maternal overprotection z=1.93, p=.05; paternal overprotection z=2.06, p=.04.		
Huyder and Nilsen, (2012)	N = 114 5- 8 years; Within-subject dyadic behavioral study	Competitive situation/structure — interpersonal; Puzzle task	Executive functioning did not predict behavior in the competitive context; better inhibitory control predicted fewer competitive behaviors only in the cooperative context.	In the cooperative-context structural model, the only significant actor path was from Simon Says inhibitory control to own competitive behavior (path coefficient ≈ −.20). In the competitive-context models, all actor and partner effects were nonsignificant, ps>.05.	Path coefficient reported graphically; exact p/CI for the significant path not numerically reported in the text.	Interpret with the following criterion-level MMAT concern in mind: Cooperative context always preceded competitive context, creating order/familiarity confounding.
Priewasser et al. (2013)	N= 71 3-5 years; Correlational behavioral study	Competitive situation/structure — strategic interpersonal competition; Bead - collecting game	False-belief understanding predicted more strategic competitive poaching moves.	False-belief group effect: H(2)=8.55, p=.014. Understanders vs non-understanders: U=115.0, z=2.95, p=.003, r=.45; medians **33.34% vs 0.00%**. False-belief score correlated with poaching, $r_s(71)=.36$, p=.002. Adding false-belief prediction improved hierarchical regression: $\Delta R^2=.046$, F(1,64)=4.57, p=.036; b=.26, p=.036.	Effect size *r* and ΔR^2 reported; CI/SE NR.	Interpret with the following criterion-level MMAT concern in mind: Local volunteer sample; some exclusions based on unusual game behavior.
Smith & LaFreniere (2013)	N = 118; 4-, 6-, and 8-year-olds; cross-sectional behavioral experiment	Strategic deception and success within interpersonal competitive game	Competitive success and strategic deception increased substantially with age; older children showed greater	Success differed by age, Wald $\chi^2(2, N = 118) = 10.2$, p = .001; success criterion reached by 11% of 4-year-olds, 49% of 6-year-olds, and 76% of 8-year-	CI / standardized effect size NR	Strong developmental gradient, but age groups were naturally occurring and the local sociodemographic sample limits generalizability.

Study	N / Design	Outcome / comparison relevant to the review	Effect direction / reported finding	Key statistical result as reported	Uncertainty / precision	MMAT-informed interpretive note
			understanding of the opponent's expectations	olds. Verbal understanding of tactical deception also differed by age, $F(2,115) = 29.9$, $p < .001$		
Strand et al. (2015)	N = 254 38–67 months; Cross-sectional comparative correlational study	Competition orientation — competitive social values; Social Orientation Choice Card	Competitive values predicted better adjustment among collectivist-background children but poorer adjustment among individualist-background children.	Multivariate SVO × culture interaction: Wilks' λ=.88, $F(12,535)=1.98$, $p=.02$. Univariate interactions: SESBI conduct problems $F(4,204)=3.13$, $p=.02$; SDQ total difficulties $F(4,204)=3.13$, $p=.02$; SDQ prosocial behavior $F(4,204)=3.99$, $p<.01$.	Adjusted means and SEs reported; CIs/effect sizes NR.	Interpret with the following criterion-level MMAT concern in mind: Restricted low-income Head Start sample limits population generalizability.
Tsiakara and Digelidis, (2015)	N =80 4–6 years; Within-subject experimental study	Competitive situation/structure — interpersonal; Build a Tower Task	Perceived performance and satisfaction were similar across the competitive and noncompetitive task contexts.	Accurate performance judgment: 47.5% **(38/80)** under NCC vs 52.5% (42/80) under competition. Happy response: 91.25% (73/80) under NCC vs 90.0% (72/80) under competition. The authors reported no significant gender/group-composition differences in construction time, satisfaction, or perceived performance.	Frequencies and percentages reported. Exact F statistics, p values, CIs, and effect sizes for the reported nonsignificant ANOVAs were **not** reported in the original article.	Interpret with the following criterion-level MMAT concern in mind: Measure reliability/validity insufficiently reported; competitive comparison also altered peer presence and comparison target.
Zhu et al. (2015)	N =321 2–6 years; Non-randomized experimental study	Competitive situation/structure — intergroup; Intergroup competition vs noncompetition condition	Intergroup competition was associated with stronger ingroup prosocial sharing among younger than older children.	Sharing game under intergroup competition: 82% egalitarian choices among 2.5–3.5-year-olds vs. 69% among 5.5–6.5-year-olds, Fisher's exact $p<.05$. Competition outcome (win vs.	Percentages, Fisher's exact tests, and Wald χ^2 statistics reported; CIs/effect sizes NR.	Interpret with the following criterion-level MMAT concern in mind: Competition/control condition not randomized and controls recruited from only three of five preschools.

Study	N / Design	Outcome / comparison relevant to the review	Effect direction / reported finding	Key statistical result as reported	Uncertainty / precision	MMAT-informed interpretive note
				loss): p=.88. Competition vs. noncompetition model: age Wald χ^2=5.735, p<.05; competition χ^2=1.002, p>.05; competition × age χ^2=1.496, p>.05. Envy game: age χ^2=6.210, p<.05; competition and competition × age nonsignificant.		
Goldvicht-Bacon & Diesendruck (2016), Exp. 2	N = 57; M = 68.14 months, SD = 6.09, range = 56–83 months; within-subject experimental task	Strategic cue selection in competition with same- vs. different-culture opponent	Children altered cue selection according to the presumed cultural knowledge of the opponent	Cue type × partner culture: F(2,55) = 5.64, p < .01, η^2 = .17. Cultural cue: different-culture M = .88, SD = .68 vs same-culture M = .44, SD = .68, t(56)=3.10, p<.005	SDs reported; CI NR	Within-subject counterbalancing strengthens internal interpretation, but the culturally homogeneous two-kindergarten sample and simulated opponent limit generalizability.
Schmidt et al. (2016)	N = 48 3-5 years; Within-subject experimental study	Competitive situation/structure — rule-governed interpersonal competition; Competitive game against a puppet varying rule adherence and game strategy	Five-year-olds, but not 3-year-olds, fully understood the dual normative structure of competitive games.	Main task four-way interaction: χ^2(1)=8.66, **p=.003**. In rule-following conditions, irrational play elicited more goal protest: χ^2(1)=8.77, p=.003, b=1.99, SE=.75, 95% CI [.52, 3.46], OR=7.30. In rule-breaking conditions, age × strategy × protest interaction **χ^2(1)=20.31, p<.001**. For 5-year-olds, strategy × protest interaction χ^2(1)=26.26, p<.001, b=6.67, SE=1.49, 95% CI [3.63, 11.08], OR=784.62.	SEs, 95% CIs and ORs reported.	Interpret with the following criterion-level MMAT concern in mind: Small local sample; otherwise strong counterbalancing and behavioral coding.

Study	N / Design	Outcome / comparison relevant to the review	Effect direction / reported finding	Key statistical result as reported	Uncertainty / precision	MMAT-informed interpretive note
Jin et al. (2017)	Exp. 1= 72 Exp. 2= 24 Exp. 3= 48 4 year; Experimental study	Competitive situation/structure — competition vs. cooperation; Fishing game	Cooperation, but not competition, improved children's reasoning about others' diverse desires.	Exp. 1: adult-gift selection: cooperation 87.5% vs competition 54.2%, χ^2=6.454, p=.011; cooperation vs individualistic χ^2=9.375, p=.002; competition vs individualistic χ^2=.333, p=.564. Exp. 3 replicated the effect: cooperation 75.0% vs competition 41.7%, between-group χ^2=5.714, p=.017.	Percentages and χ^2/p reported; CIs/effect sizes NR.	Interpret with the following criterion-level MMAT concern in mind: Random assignment reported, but sequence generation/concealment and assessor blinding insufficiently described.
Mui et al. (2017)	55 Chinese and 31 Dutch 8-year-olds; Experimental cross-cultural stud	Competitive outcomes/experiences — win/loss within structured competition; Competitive number-guessing game	Children's smiles following winning and losing varied as a function of social and cultural context. Duchenne smiles occurred more frequently in paired play, and the effect of sociality on smile intensity was more pronounced among Chinese than Dutch children.	Smile type × social context: $\chi^2(1)$=8.29, p=.004, φ=.30, OR=3.59. Overall smile intensity: social-context main effect F(1,58)=13.79, p<.001, ηp^2=.19 and social × culture interaction F(1,58)=13.75, p<.001, ηp^2=.19. Chinese paired vs alone difference Mdiff=3.41, SD=1.24, p<.001; Dutch Mdiff=.002, SD=1.03, **p=1.00**. Win/loss was unrelated to smile presence, McNemar $\chi^2(1)$=.063, p=.80, or smile type, $\chi^2(1)$=1.23, p=.27.	ηp^2, φ, OR, means/SDs reported; CI NR.	MMAT-informed caution: Social condition was randomized, but cultural group was naturally occurring at the school level and paired children selected their own friends, leaving contextual confounding.
Pappert et al. (2017)	N = 50 4-6 years; Randomized between-subject experimental study	Prosocial resource allocation following competitive vs. noncompetitive coloring context	Competition reduced prosocial responding specifically when children were disadvantaged and choosing the	Inequality type × cost × condition: F(1,47) = 4.19, p = .046, ηp^2 = .08. Costly disadvantageous inequality: competition M =	95% CIs and standardized effect size reported	Competition effect was condition-specific rather than general across resource-allocation decisions.

Study	N / Design	Outcome / comparison relevant to the review	Effect direction / reported finding	Key statistical result as reported	Uncertainty / precision	MMAT-informed interpretive note
			prosocial option entailed a cost; no condition differences emerged for the other trial types.	1.92, 95% CI [1.30, 2.59] vs. control M = 2.96, 95% CI [2.45, 3.42], t(44) = −2.42, p = .020, d = .73. Other trial types: all ts < 1.		
Domberg et al. (2018)	N= 240 5-7 years; Randomized between-subject experimental studies	Competitive situation/structure — competition vs. cooperation; Zoo Game	Children produced fewer and more self-serving arguments in competitive than cooperative contexts, particularly at age 7.	Study 1: full model χ^2=24.74, df=7, p<.001; age × condition × argument-type interaction χ^2=5.30, df=1, p=.021. Seven-year-olds used fewer trained arguments in competition than cooperation in Study 2, F(1,33)=7.41, p=.010, ηp^2=.18; overall condition effect F(1,67)=4.44, p=.039, ηp^2=.06. Five-year-olds showed no condition difference, F(1,33)=.25, p=.619.	ηp^2 reported for Study 2; no CI reported for these effects.	Interpret with the following criterion-level MMAT concern in mind: Random assignment reported, but generation/concealment and assessor blinding unclear.
Hu & Zhu (2018)	N = 166 recruited; final analytic N = 163; ages 2–6 years; experimental study	Age and willingness to compete again	Younger children were more likely than older children to choose a subsequent competition.	Rival: β = −1.252, p = .036, OR = .286; χ^2(1, N = 163) = 4.725, p = .030. Non-rival: β = −1.513, p = .010, OR = .220	OR and p values reported; CI/SE NR.	Interpret with the following criterion-level MMAT concern in mind: Win/loss reflected actual performance and was therefore confounded with ability/effort.
Herrmann et al. (2019)	Study 1 N= 128 Study 2 N= 64 5-8 years; Experimental	Competitive situation/structure — competitive partner-choice; Competitive altruism dyadic sharing game	Eight-year-olds increased prosocial sharing when being observed by a peer who could later select them as a partner, indicating	Study 1: full-null model χ^2=10.55, df=3, p=.014; choice × observer interaction estimate=−.520, SE=.188, χ^2=6.190, p=.013. Competitive altruism > audience p=.002; > control	Model estimate, SE and exact p values reported; CIs not reported in text.	MMAT-informed caution: Random assignment was reported, but Study 1 had notable post-assignment exclusions and outcome-assessor blinding was not reported clearly.

Study	N / Design	Outcome / comparison relevant to the review	Effect direction / reported finding	Key statistical result as reported	Uncertainty / precision	MMAT-informed interpretive note
			strategic reputation management in competitive partner-choice contexts. Five-year-olds did not show the same competitive-altruism effect.	choice p=.013; > control p=.049. Condition × observer × trial interaction χ^2=6.19, p=.013. Study 2, 5-year-olds: condition effect nonsignificant, estimate=.08, SE=.13, χ^2=.38, p=.536.		
P. Li et al. (2019)	Exp 1 =48 Exp 2 =72 4–5 years; Randomized experimental study	Competitive situation/structure — competition vs. cooperation; Fishing Game	Cooperation, but not competition, improved flexible visual perspective taking	Exp. 2: condition effect F(2,66)=7.18, p<.01. Cooperation > competition p=.004 and > baseline p=.001; competition vs baseline p=.603. Age main effect F(1,66)=1.01, p=.32 and age × condition interaction F(2,66)=1.50, p=.23 were nonsignificant.	Exact post-hoc p values reported; means/CI/effect size for the omnibus effect not reported in the article text.	Interpret with the following criterion-level MMAT concern in mind: Post-allocation exclusions transparent; randomization procedure and assessor blinding incompletely reported.
Toppe et al. (2019)	N = 96 4-6 years; Randomized experimental	Sharing with uninvolved third party; social inclusion; subsequent free-play prosociality	Children shared more after cooperative than competitive play; solitary play was intermediate. No condition effect emerged for social inclusion or free-play prosociality.	Gaming context effect on sharing: $\chi^2(2)$ = 6.004, p = .050. Cooperative: M = 4.33, SD = 1.14; competitive: M = 2.90, SD = 2.15; solitary: M = 3.55, SD = 2.22. Competitive relative to cooperative: estimate = −.407, SE = .166 (equivalent reverse-coded contrast in Table 1: .407, SE = .166, p = .014).	SDs and model SE reported; CI NR	Random assignment and blinded assessment of the dictator game strengthen interpretation, although the study was below its preregistered target sample size.
Castellano-Navarro	N= 48 6-year-olds; Randomized	Competitive situation/structure — competition vs. cooperation; reward-allocation context; Competitive vs cooperative reward-allocation context	Six-year-olds performed better in com-	For child–child dyads in Experiment 1, competition > coopera-	Exact U and p reported; group means/SDs are	Interpret with the following criterion-level MMAT concern in mind: Strong blinded coding,

Study	N / Design	Outcome / comparison relevant to the review	Effect direction / reported finding	Key statistical result as reported	Uncertainty / precision	MMAT-informed interpretive note
et al. (2020)	experimental study		petition than cooperation on visual-perspective inference.	tion for visual-perception performance: Mann–Whitney U = 40.50, n = 24, p = .034. Children did not show successful performance on the knowledge and false-belief tasks, so those conditions were not used in the competition–cooperation comparison.	presented graphically, but not numerically in the text.	but randomization sequence/concealment not described.
Roberts et al. (2020)	N = 92 4–5 years; Vignette-based quantitative study	Competitive situation/structure — hypothetical resource competition; Hypothetical resource-competition vignettes	Girls chose more prosocial and boys more coercive strategies, with responses also varying by opponent toughness.	Toughness effect on coercive responses: boys $F(1,40)=4.73$, $p=.036$, $\omega^2=.08$, very tough $M=3.93$, $SD=2.64$ vs not tough $M=3.45$, $SD=2.67$; girls $F(1,41)=5.21$, $p=.028$, $\omega^2=.09$, $M=1.74$, $SD=2.54$ vs 1.30, $SD=2.23$. For boys, coercive responding declined with age: $F(1,40)=6.26$, $p=.017$, $\omega^2=.11$.	Means/SDs and ω^2 reported; CIs NR.	Interpret with the following criterion-level MMAT concern in mind: Local sample; adapted hypothetical vignette measure lacked reported reliability/coder agreement.
Sai et al. (2020)	N = 97 3–4 years; Cross-sectional correlational behavioral study	Competitive situation/structure — strategic interpersonal competition; Competitive hide-and-seek game	Competitive-game lying was associated with aspects of mental-state understanding, particularly diverse-belief and belief-emotion understanding, whereas concealing-transgression lying	Competitive-lying frequency correlated with knowledge access $r=.23$, $p=.025$, belief-emotion $r=.29$, $p=.005$, and verbal ability $r=.22$, $p=.032$. After controlling age/language, belief-emotion remained associated, $r=.27$, $p=.009$. Final regression block: $\Delta F(3,91)=4.64$, $\Delta R^2=.122$, $p=.001$; diverse belief $p=.045$,	Correlations, ΔR^2 and Nagelkerke R^2 reported; CIs generally NR.	Interpret with the following criterion-level MMAT concern in mind: Two-preschool sample; correlational design prevents causal interpretation.

Study	N / Design	Outcome / comparison relevant to the review	Effect direction / reported finding	Key statistical result as reported	Uncertainty / precision	MMAT-informed interpretive note
			showed no comparable associations.	belief-emotion p=.008. Dichotomous lying model: $\chi^2(5,97)=18.80$, Nagelkerke $R^2=.235$, p=.002.		
Eriksson et al. (2021)	N = 65 4-5 years; Between-subject 6-week experimental	Behavior after cooperative vs. competitive board games	Competitive-board-game exposure was followed by more competitive behavior; prosocial and antisocial behavior did not differ significantly.	Competitive behavior: t(58) = −2.90, p = .005, d = .77; cooperative M = .18, competitive M = .31. Prosocial behavior: p = .632; antisocial behavior: p = .333	Effect size (d) and p values reported; CI/SE NR.	Interpret with the following criterion-level MMAT concern in mind: Variable intervention exposure/task completion and very low reliability for competitive-behavior coding.
Proft et al. (2021)	N=81 3 years; Randomized mixed-design experimental	Competitive situation/structure — competitive goal relation; Competitive vs incompatible-desire payoff condition	Three-year-olds could ascribe competitive desires but struggled to translate this understanding into strategic choices.	Choice/action performance in competition did not exceed chance: t(38) = −.85, p = .401, r = .14, BF_{01} = 5.65. Children chose the self-favoring option more often in incompatible-desire than competition trials, t(77)=2.85, p=.006, r=.31. Explicit-question ANOVA: condition F(1,78)=6.72, p=.011, ηp^2=.08; type of question F(1,78)=11.02, p=.001, ηp^2=.12; interaction F(1,78)=2.03, p=.158, ηp^2=.03, BF_{01}=1.94.	Effect sizes and Bayes factors reported; CI NR.	Interpret with the following criterion-level MMAT concern in mind: Randomization procedure, baseline equivalence, assessor blinding, and adherence incompletely documented.
Reyes-Jaquez & Echols (2021)	N = 88 3–4 years; Randomized between-subject experimental	Competitive situation/structure — competition vs. cooperation; reward structure; Candy-finding game	Children endorsed partners' claims more under cooperative	Round 2: reward-rules × D-N interaction B=−.44, SE=.18, Wald χ^2=5.98, p=.014, Exp(B)=.64, 95% CI [.45,.92]. In	B, SE, OR/Exp(B), 95% CIs and	Interpret with the following criterion-level MMAT concern in mind: Experimenter adminis-

Study	N / Design	Outcome / comparison relevant to the review	Effect direction / reported finding	Key statistical result as reported	Uncertainty / precision	MMAT-informed interpretive note
			than competitive reward rules; stronger EF supported rule-consistent responses, whereas ToM was unrelated.	high-EF children, common vs conflicting rules: B=−2.59, SE=.50, Wald χ^2=26.99, p<.001, Exp(B)=.08, 95% CI [.03,.20]; acceptance M=.94, SE=.03 vs M=.54, SE=.07. Lower-EF children showed no rule effect, p=.336.	means/SEs reported.	tered the condition and recorded behavioral responses; assessor was not blinded.
Toppe et al. (2021)	N= 144 4-6 years; Randomized between-dyad experimental	Competitive situation/structure — competitive vs. cooperative vs. solitary play; Cooperative, competitive, or solitary game	Gaming context did not affect later prosociality, although competition increased physical engagement during play.	Engagement: condition × gaming-phase interaction χ^2(2)=32.081, p<.0001; increase in tube rotations: competition Δ=9.273, cooperation Δ=1.882, solitary Δ=4.924. Sharing full-null comparison: χ^2(17)=15.471, p=.562, BF<.001. Social inclusion models all null: general inclusion χ^2(11)=14.261, p=.219; first inclusion χ^2(11)=4.541, p=.951; passes χ^2(11)=12.561, p=.323; directives χ^2(11)=10.345, p=.500, all BFs<.0001.	Exact χ^2/p and Bayes factors reported; descriptive sharing means/SDs available.	Interpret with the following criterion-level MMAT concern in mind: Preregistered observer baseline excluded from inferential analyses; condition blinding not explicit.
Tsoi et al. (2021)	N =120 4 year; Between-subject experimental	Competitive situation/structure — competition vs. cooperation; Stickers Task	Four-year-olds were better at inducing false beliefs in cooperation than competition.	Stickers task omnibus: χ^2(2)=14.964, p<.001, Tjur R^2=.056. Condition × age: χ^2(1)=5.884, p=.015; GLMM replication χ^2(1)=6.78, p=.009. Trialwise interaction remained, χ^2(1)=6.947, p=.008. Memory:	Likelihood-ratio χ^2, Tjur R^2, exact p values; Figure reports 95% CIs.	Interpret with the following criterion-level MMAT concern in mind: Convenience recruitment and unclear allocation procedure; alternative explanations were only partly controlled.

Study	N / Design	Outcome / comparison relevant to the review	Effect direction / reported finding	Key statistical result as reported	Uncertainty / precision	MMAT-informed interpretive note
				condition × age $\chi^2(1)=1.66$, p=.20; Day-Night EF task: $\chi^2(1)=.473$, p=.49; condition main effect $\chi^2(1)=2.57$, p=.11.		
M. Zhang and Sylva (2021)	N = 68 3–4 and 6–7 years; Sequential within-subject experimental	Competitive situation/structure — intergroup; Noncompetitive followed by intergroup competitive context	Competition increased older children's reliance on ingroup informants; younger children showed no clear shift.	Direct context effect on testimony endorsement: $F_{ATS}(1,\infty)=8.91$, p=.003. Older children endorsed ingroup informants more under competition at baseline, z=−2.00, p=.045, r=−.35, and consistent condition, z=−2.07, p=.039, r=−.36; no context effect in inconsistent condition. Person judgment: older children changed choices across contexts in inconsistent condition, Wald $\chi^2(1)=6.04$, p=.014; competitive vs noncompetitive OR for all children 1.82, p=.041.	Effect-size r, ORs, 95% CIs in GEE model, and exact p values reported.	Interpret with the following criterion-level MMAT concern in mind: Noncompetitive condition always preceded competition; carryover/order effects cannot be separated.
Bayındır et al. (2022)	N =260 4-6 years; Cross-sectional correlational	Competition orientation — task-oriented vs. other-referenced; PCQ	Better self-regulation predicted more task-oriented competition, while control skills predicted less other-referenced competition.	Task-oriented: regulation β=.39, p<.01, control β=.47, p<.01; self-regulation variables added 35% variance, F(2,256)=73.75, p<.001, R^2=.63. Regulation × gender β=−.13, p<.05; slopes: boys .65, t=8.08, p<.001, girls .27, t=2.87, p<.01. Other-referenced:	Standardized βs, $R^2/\Delta R^2$, t and p values reported; CIs NR.	Interpret with the following criterion-level MMAT concern in mind: Same teachers rated regulation and competition, creating common-rater bias.

Study	N / Design	Outcome / comparison relevant to the review	Effect direction / reported finding	Key statistical result as reported	Uncertainty / precision	MMAT-informed interpretive note
				regulation β=.49, p<.01, control β=−.73, p<.01; ΔR^2=.25, R^2=.47.		
Fish et al. (2023)	N = 274 5–8; Randomized between-subject experimental	Competitive situation/structure — competition vs. collaboration vs. solo; Proportion-learning game under competition, collaboration, or solo conditions	Competition increased perseverance and task engagement, but older boys showed poorer proportional reasoning under competition.	Conflict trials: condition × gender × grade B=.42, t=2.74, p=.007, BF=5.24. Second-grade gender × competition B=−.36, t=−3.10, p=.002. For boys, competition effect B=−.22, t=−2.57, p=.013. Second-grade boys: competition EMM=.55, SE=.06 vs baseline .77 (.06), p=.025, BF_{10}=4.17 and collaboration .76 (.06), p=.025, BF_{10}=2.49. Simple trials: all model effects ps>.086, null model BF=12.56.	EMMs/SEs, regression coefficients, exact p values and Bayes factors reported.	Interpret with the following criterion-level MMAT concern in mind: Stratified random assignment reported, but sequence/concealment and assessor blinding insufficiently detailed.
Özçelik and Sapsağlam, (2023)	N =287 4-6 years; Cross-sectional correlational	Competition styles and social competence/behavior	Task-oriented competition was positively related to social competence, whereas other-referenced competition was positively related to anger-aggression.	Task-oriented competition × social competence: r = .719, p < .01; other-referenced competition × anger-aggression: r = .582, p < .01; task-oriented competition × anger-aggression: r = −.372, p < .01	p values reported; CI/SE NR.	Interpret with the following criterion-level MMAT concern in mind: Nonprobability sampling and primarily bivariate analyses without adjusted confounder models.
Prodan et al. (2023)	N = 75, ages 6–8 years; Non-randomized quantitative behavioral study	Competitive situation/structure — strategic interpersonal competition; Computerized competitive hide-and-seek game	Children deceived familiar peers less than unfamiliar peers; ToM (Theory of Mind) and executive functions were associated with	Familiarity effect: F(2.30,74)=4.69, p=.007, ηp^2=.06. Liked-card deception: unfamiliar M=.784, SD=.247 vs familiar M=.675, SD=.245. Overall socio-cognitive model:	Means/SDs, ηp^2, βs, adjusted R^2 and 95% CIs reported.	Interpret with the following criterion-level MMAT concern in mind: Single urban, middle-to-high-income school sample limits representativeness; the novel computerized deception

Study	N / Design	Outcome / comparison relevant to the review	Effect direction / reported finding	Key statistical result as reported	Uncertainty / precision	MMAT-informed interpretive note
			strategic deceptive performance.	$F(8,291)=18.95$, adjusted $R^2=.32$, $p<.001$. First-order FB $\beta=.08$, 95% CI [.02,.14], $p=.012$; second-order FB $\beta=.12$ [.06,.18], $p<.001$; visuospatial WM $\beta=.02$ [.01,.04], $p=.004$. Inhibitory control main effect was null, $\beta=.02$ [−.03,.06], $p=.483$.		task and partially unbalanced familiarity manipulation constrain generalizability and the scope of familiarity comparisons.
Savage et al. (2023)	Final N = 211 43–66 months; Cross-sectional behavioral study	Competition orientation — competitive social-value orientation; Competitive-versus-cooperative resource-allocation choice	Competitive social values differed by family cultural background; English-speaking Latinx children were most likely to make competitive rather than cooperative choices. However, social-value orientation and its interaction with cultural background were unrelated to teacher-rated prosocial or problem behavior.	English-speaking Latinx children chose competitively more often (49%) than the combined comparison group (32%), $\chi^2(1)=6.18$, $p<.05$, Cramer's $V=.17$; vs Spanish-speaking Latinx children (30%), $\chi^2(1)=5.54$, $p<.05$, $V=.19$. Behavioral adjustment: SVO Wilks' $\lambda=.95$, $F(4,328)=2.21$, $p=.07$; culture $\lambda=.97$, $F(4,328)=1.13$, $p=.34$; SVO × culture $\lambda=.97$, $F(8,328)=.65$, $p=.73$.	Percentages, Cramer's V, Wilks' λ and exact p values reported; CIs NR.	MMAT-informed caution: Head Start sample with substantial exclusions, including 42 children who failed the SVO understanding check; cultural-group comparisons also rely on demographic classification.
Uyanık et al. (2023)	N =202 4-6 years; Cross-sectional correlational	Play skills and competition styles	Better play skills were associated with less other-oriented and more task-oriented competition.	Total play skills × other-oriented competition: $r = -.358$, $p < .01$; total play skills × task-oriented competition: $r = .357$, $p < .01$	p values reported; CI/SE NR.	Interpret with the following criterion-level MMAT concern in mind: Convenience sample and unadjusted bivariate analyses.

Study	N / Design	Outcome / comparison relevant to the review	Effect direction / reported finding	Key statistical result as reported	Uncertainty / precision	MMAT-informed interpretive note
Valcke and Nilsen, (2023)	N = 118 4-6 years; Within-subject repeated-measures experimental	Competitive vs. cooperative task goals and antagonistic verbal responses	Competitive task goals elicited more antagonistic verbal responses, particularly following self-oriented messages.	Task goal: $\beta = .61$, $t(322) = 7.94$, $p < .001$, 95% CI [.46, .77]; task goal × message: $\beta = .08$, $t(351) = 2.24$, $p = .03$, 95% CI [.01, .16]	95% CI and p values reported.	Interpret with the following criterion-level MMAT concern in mind: Local consent-based sample; fictional partners improve control but reduce ecological authenticity.
Xiao et al. (2023)	N = 374; 5–8 years; between-subject experimental study	Competitive situation/structure — intergroup competition; win vs. loss vs. noncompetition; intergroup familiarity; merit-based resource allocation	Children allocated more to their own group after winning and less after losing than in the noncompetitive condition. Familiarity was associated with less own-group bias among 5–6-year-olds, but not 7–8-year-olds.	Condition: $F(2,362) = 105.92$, $p < .001$, $\eta p^2 = .37$; win M = 2.48, noncompetition M = 2.11, loss M = 1.50. Age × familiarity: $F(1,362) = 7.47$, $p = .007$, $\eta p^2 = .02$.	Effect sizes (ηp^2), p values, means, and SDs reported; CI NR.	Interpret with caution: single-region Han Chinese sample limits representativeness; win/loss reflected actual performance and familiarity partly reflected naturally occurring friendship relations, limiting causal attribution of outcome-status and familiarity effects.
Grueneisen et al. (2024)	Exp. 1: n=69; Exp. 2: n=120; total N=189; 4–7 years; Two within-subject experimental studies	Competitive situation/structure — competitive partner-choice; Cooperative vs competitive partner-choice tasks	From about age 5, children selected task-relevant partners for cooperation but strategically avoided them as competitors.	Exp. 1: age × condition $\chi^2(1)=11.07$, $p<.001$; 6–7-year-olds selected task-relevant partners 61% in cooperation vs 31% in competition, $\chi^2(1)=18.70$, $p<.001$; no condition effect for 4–5-year-olds, $\chi^2(1)=1.15$, $p=.283$. Exp. 2 replicated age × condition, $\chi^2(1)=8.37$, $p=.004$; 6–7-year-olds: $\chi^2(1)=31.60$, $p<.001$; 5-year-olds differentiated conditions, $\chi^2(1)=10.07$, **$p=.002$**,	Figures report 95% CIs around partner-choice probabilities; likelihood-ratio χ^2 and exact p values reported. Model-coefficient CIs are not reported in the main-text results extracted here.	Interpret with the following criterion-level MMAT concern in mind: Participant-database/local recruitment and hypothetical partner-choice tasks constrain generalizability.

Study	N / Design	Outcome / comparison relevant to the review	Effect direction / reported finding	Key statistical result as reported	Uncertainty / precision	MMAT-informed interpretive note
				whereas 4-year-olds did not, $\chi^2(1)=0.00$, p=.952.		
Yıldız and Sak, (2024)	N = 400 4-6 years; Cross-sectional correlational survey study	Competition styles and anxiety	Higher task-oriented competition was weakly associated with lower anxiety; other-referenced competition was weakly negatively related to separation anxiety.	Task-oriented competition: generalized anxiety r = −.159, p < .01; separation anxiety r = −.138, p < .01; specific phobia r = −.102, p < .05; overall anxiety r = −.156, p < .01. Other-referenced competition × separation anxiety: r = −.109, p < .05	p values reported; CI/SE NR.	Interpret with the following criterion-level MMAT concern in mind: Probability sampling was strong, but relationships were based on bivariate correlations without multivariable confounder adjustment.
Xiao, Zhang, & Li, (2024)	Study 1: n=208; Study 2: n=280; Study 3: n=250; ages 3–8 years; Three experimental studies	Interpersonal vs. intergroup competition and merit-based allocation	Children allocated more to their own side after interpersonal than intergroup competition under win and loss conditions.	Competition size × win: B = −1.26, SE = .49, t = −2.59, p = .010, 95% CI [−2.22, −0.30]; interpersonal > intergroup own-side allocation after win p < .001 and loss p = .006	SE, 95% CI, and p values reported.	Interpret with the following criterion-level MMAT concern in mind: Win/loss was performance-determined; interpersonal/intergroup comparisons also relied on separate samples.
Xiao, Wang and Li, (2024)	N = 310 5-6 and 7-8 year; Between-subject experimental study	Competitive situation/structure — intergroup; resource-allocation context; Finding football competition	Between-group competition shifted older children toward more equal allocations with ingroup collaborators, whereas younger children remained merit-oriented.	Interpersonal: win B=.78, SE=.37, p=.036, 95% CI [.05,1.52]; loss B=1.01, SE=.35, p=.005, CI [.31,1.71]; age × loss B=−.19, SE=.06, p<.001, CI [−.30,−.08]. Intergroup: age × win B=.12, SE=.05, p=.017, CI [.02,.22]; age × loss B=−.12, SE=.05, p=.016, CI [−.23,−.02]. Combined interpersonal/intergroup analysis: competition-size × win B=−1.26, SE=.49,	B, SE, 95% CIs, exact p values, simple slopes and Johnson–Neyman thresholds reported.	Interpret with the following criterion-level MMAT concern in mind: Noncompetition and merit were randomized, but actual competitive win/loss remained performance-determined.

Study	N / Design	Outcome / comparison relevant to the review	Effect direction / reported finding	Key statistical result as reported	Uncertainty / precision	MMAT-informed interpretive note
				t=−2.59, p=.010, 95% CI [−2.22,−.30].		
Çantaoğlu et al. (2025)	N = 337 4–6 years; Cross-sectional relational survey study	Digital-game addiction dimensions and competition styles	Digital-game addiction indicators showed weak positive associations with other-oriented competition; task-oriented competition was also positively related to dominance maintenance.	Other-oriented competition × willingness: r = .202, p < .001; other-oriented competition × blocking: r = .116, p < .05; task-oriented competition × dominance-maintenance: r = .296, p < .001	p values reported; CI/SE NR.	Interpret with the following criterion-level MMAT concern in mind: Purposive sample; current-sample reliability insufficiently reported for one principal measure; analyses unadjusted.
Priewasser et al. (2025)	N = 51 34–56 months; Within-subject experimental	Competitive situation/structure — strategic competition/sabotage; Cooperative and competitive sabotage-story scenarios	Children succeeded in cooperative scenarios but struggled with competitive sabotage; sabotage understanding was associated with false-belief understanding.	Competition score M=52.4%, SD=45.7 vs cooperation M=90.2%, SD=30.0; each competitive test differed from cooperation, McNemar p≤.002. Competition performance varied with false-belief score, H(3)=10.575, p=.014, r=.22; ordered trend J=650.5, z=2.99, p=.003, r=.37. Competition–false belief correlation r(51)=.43, p=.002; adjusted for age/general abilities r(44)=.35, p=.016; additionally controlling cooperation r(43)=.33, p=.028.	Means/SDs, correlation coefficients, rank-based effect sizes r, and exact p values reported; CIs NR.	Interpret with the following criterion-level MMAT concern in mind: 18/69 children excluded, including comprehension/familiarization failures, creating potentially selective attrition.
Xiao & Li (2025)	Study 1: N = 298; Study 2: N = 264;	Competitive situation/structure — intergroup; secondary feature: win/loss; Passing ball game	With increasing age, children made more equal allocations to in-	Study 1: model R²=.10, F(6,291)=5.44, p<.001; loss B=.59, SE=.22, p=.007, 95% CI	Regression SEs and 95% CIs reported in Study	MMAT-informed caution: Noncompetition versus competition was assigned, but

Study	N / Design	Outcome / comparison relevant to the review	Effect direction / reported finding	Key statistical result as reported	Uncertainty / precision	MMAT-informed interpretive note
	3–8 years; Experimental, two-study design; between-group competition		group collaborators after winning but more generous allocations after losing. The findings suggest that children balance fairness concerns and in-group relational concerns differently according to competitive group status.	[.16,1.02]; age × loss B=−.11, SE=.04, p=.002, CI [−.18,−.04]; loss simple slope −.13, t=−5.39, p<.001. Generous vs equal allocations under loss increased with age, B=1.02, SE=.33, OR=4.13, p=.016. Study 2: unequal advantageous allocations were increasingly rejected with age in win (slope=.83, SE=.12, p<.001), loss (.46, SE=.12, p<.001) and noncompetition (.23, SE=.10, p=.028); win slope exceeded loss, difference=.40, SE=.16, p=.035, and noncompetition, difference=.63, SE=.16, p<.001.	1; Study 2 reports SEs, exact p values and Johnson–Neyman thresholds; figures display 95% CIs.	win/loss status was performance-determined, limiting causal interpretation of outcome-status effects.
Q. Zhang et al. (2025)	N =120 4–5 years; Randomized between-subject experimental study	Competitive vs. cooperative game setting and sharing	Cooperative game settings produced higher sharing attitudes and sharing behavior than competitive settings.	Sharing attitude: $F(1,115) = 4.65$, $p = .03$, $d = .40$, $\eta p^2 = .04$; cooperative M = .82, competitive M = .65. Sharing behavior: $F(1,115) = 5.72$, $p = .02$, $d = .44$, $\eta p^2 = .05$; cooperative M = 2.42, competitive M = 1.88	Effect sizes (d, ηp^2) and p values reported; CI/SE NR.	Interpret with the following criterion-level MMAT concern in mind: Baseline comparability and assessor blinding incompletely reported.
Zhao et al. (2025)	N = 601 3–8 years; Randomized between-subject experimental study	Age, interaction type, social context, and resource allocation	The effect of interindividual vs. intergroup interaction on allocation varied by age and	Age × interaction type: $F(1,589) = 4.50$, $p = .034$, $\eta p^2 = .008$; age × interaction type × social context: $F(2,589) = 5.38$, $p = .005$, $\eta p^2 = .018$	Effect size (ηp^2) and p values reported; CI/SE NR.	Interpret with the following criterion-level MMAT concern in mind: Large balanced sample, but randomization procedure

Study	N / Design	Outcome / comparison relevant to the review	Effect direction / reported finding	Key statistical result as reported	Uncertainty / precision	MMAT-informed interpretive note
			by cooperative/competitive/neutral context.			and condition-blinding not fully reported.

Note 1. This table supplements the narrative study-level findings reported in Table 1 of the main manuscript. Only review-relevant statistical results that were explicitly reported in an extractable numerical form in the available study reports are summarized here. Statistics are reproduced in the form reported by the original authors; no recalculation, standardization, or transformation was performed. Qualitative findings and quantitative studies for which the relevant result was not reported in a directly extractable numerical form remain summarized narratively in Table 1.

Note 2. Pappert et al. (2017) additionally reported an effect of condition on crayon sharing as $F(2,45) = 4.39$, $p = .042$, $\eta p^2 = .089$. Because these statistics are internally inconsistent in the published article, this test was not corrected by inference and was not used as the principal numerical estimate in the synthesis
